# CRAT downregulation promotes ovarian cancer progression by facilitating mitochondrial metabolism through decreasing the acetylation of PGC-1α

**DOI:** 10.1038/s41420-025-02294-2

**Published:** 2025-01-19

**Authors:** Zhen Zhang, Shuhua Zhao, Xiaohui Lv, Yan Gao, Qian Guo, Yanjie Ren, Yuanyuan He, Yihua Jin, Hong Yang, Shujuan Liu, Xiaohong Zhang

**Affiliations:** 1 Department of stomatology, Shaanxi Provincial People’s Hospital, Xi’an, China; 2 Department of Gynaecology and Obstetrics, Xijing Hospital, Air Force Medical University, Xi’an, China

**Keywords:** Ovarian cancer, Ovarian cancer

## Abstract

Mitochondrial dysfunctions are closely associated with different types of disease, including cancer. Carnitine acetyltransferase (CRAT) is a mitochondrial-localized enzyme catalyzing the reversible transfer of acyl groups from an acyl-CoA thioester to carnitine and regulates the ratio of acyl-CoA/CoA. Our bioinformatics analysis using public database revealed a significant decrease of CRAT expression in ovarian cancer (OC). However, the functions of CRAT have rarely been investigated in human cancers, especially in OC. Here, we found a frequent down-regulation of CRAT in OC, which is mainly caused by up-regulation of miR-132-5p. Downregulation of CRAT was significantly associated with shorter survival time for patients with OC. Forced expression of CRAT suppressed OC growth and metastasis by inducing cell cycle arrest and epithelial to mesenchymal transition (EMT). By contrast, CRAT knockdown promoted OC growth and metastasis. Mechanistically, we found that CRAT downregulation promoted OC growth and metastasis by increasing mitochondrial biogenesis to facilitate mitochondrial metabolism through reducing the acetylation of peroxisome proliferator-activated receptor-γ coactivator (PGC-1α). In summary, CRAT functions as a critical tumor suppressor in OC progression by enhancing PGC-1α-mediated mitochondrial biogenesis and metabolism, suggesting CRAT as a potential therapeutic target in treatment of OC.

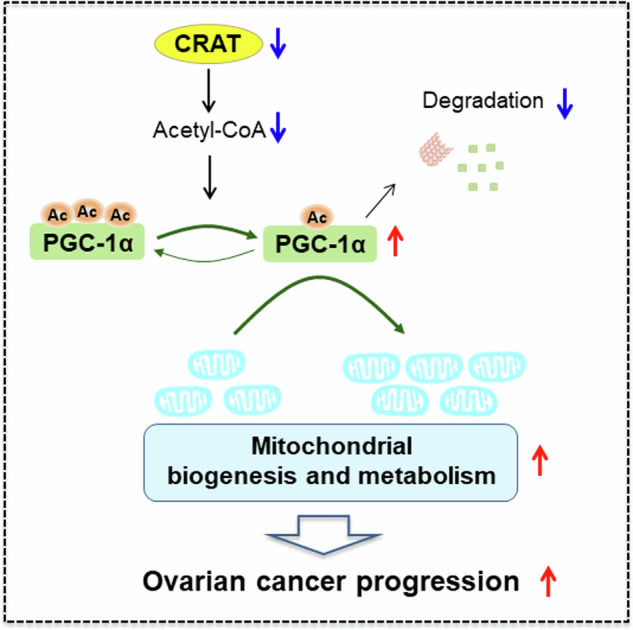

## Introduction

Mitochondria play a pivotal role in regulating energy production, metabolic signaling and oxidative stress, and their dysfunction is closely linked to the pathogenesis of multiple human diseases, including cancer [1]. Cumulating evidence has indicated that mitochondrial metabolism supports tumor progression not only by serving as a major source of ATP, but also by providing key metabolites for macromolecule synthesis and signaling transduction during tumorigenesis [2]. Thus, mitochondria metabolism now has become an attractive target for cancer therapy [3]. While cumulative evidence has demonstrated that mitochondrial metabolism reprogramming plays crucial roles in the promotion of cancer progression, the molecular mechanisms underlying the alterations of mitochondrial metabolism in cancer cells remain incompletely understood.

CRAT is localized primarily in the mitochondrial and functions as an enzyme catalyzing the reversible transfer of acyl groups from an acyl-CoA thioester to carnitine [4]. In addition to its localization in mitochondria, CRAT has also been reported to present in several other subcellular regions, such as cytoplasm [5]. It has been demonstrated that acetylcarnitine produced by mitochondrial CRAT can be shuttled out of the mitochondria to produce cytosolic acetyl-CoA by means of a cytosolic CRAT [5]. Studies have demonstrated a significant role of CRAT in several types of metabolic disorders such as obesity and diabetes [6–8]. It was also reported that CRAT deficiency-induced mitochondrial dysfunction was closely associated with cellular senescence and aging [9]. These findings suggest that CRAT may play an important role in the occurrence and progression of a variety of metabolic-associated diseases. However, the expression pattern and functions of CRAT remain largely unexplored in human cancers, especially in OC. Our bioinformatics analysis using public high-throughput database revealed a significant downregulation of CRAT in tumor tissues of ovarian cancer (OC), as compared to corresponding normal tissues, implying that CRAT may play a role in the progression of OC.

In this study, we investigated the expression and clinical significance of CRAT in OC, especially focused on its biological functions in metabolism and progression of OC. We found that CRAT functions as a critical tumor suppressor during OC progression by enhancing PGC-1α-mediated mitochondrial biogenesis and metabolism.

## Results

### CRAT expression was down-regulated in OC and its downregulation was correlated with poor patient survival

**Fig. 1:**
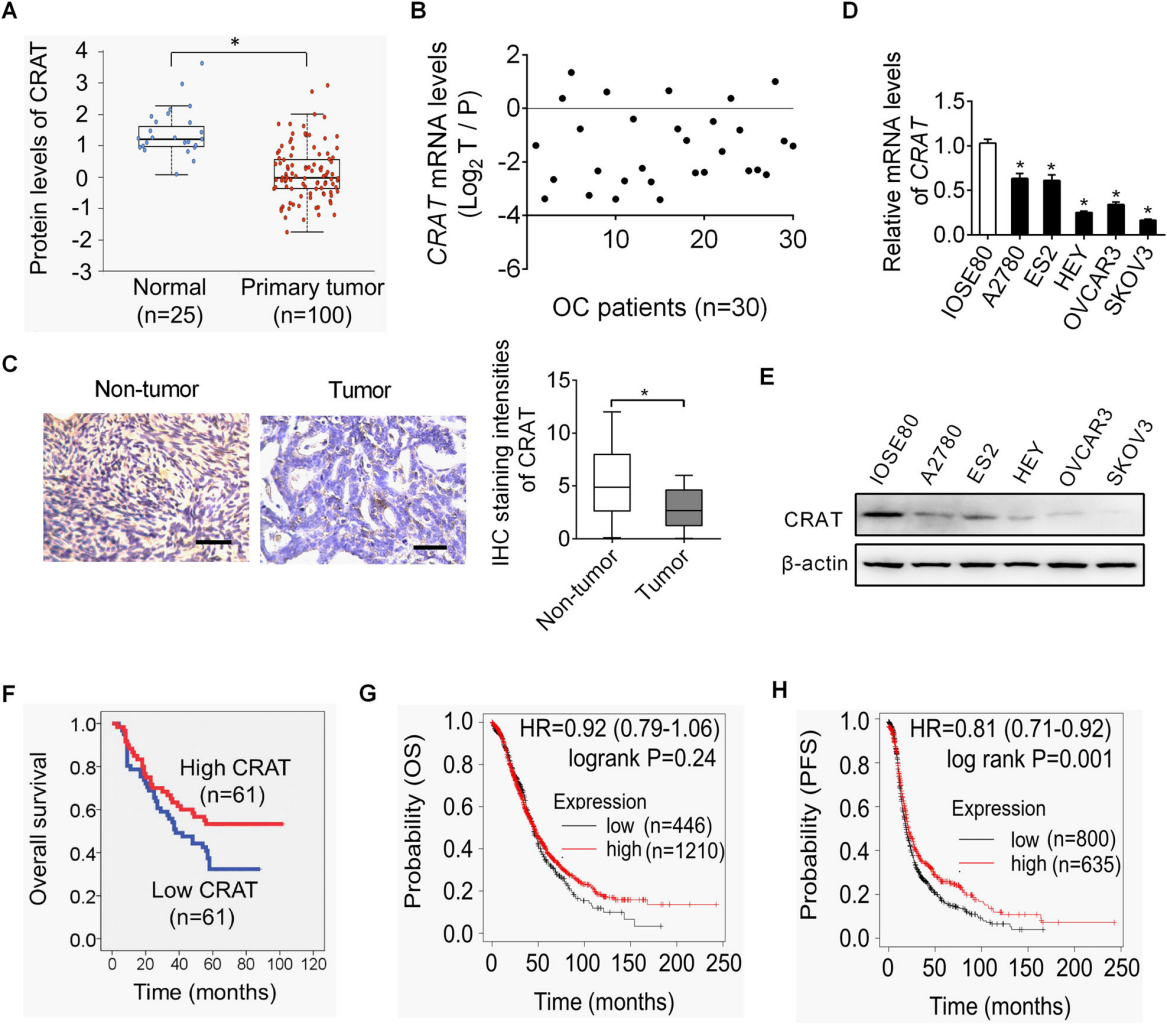
CRAT expression was down-regulated in OC and its downregulation was correlated with poor patient survival. **A** CRAT expression at protein level was analyzed by bioinformatics analysis in the TCGA dataset of ovarian cancer (OC). **B** CRAT expression was analyzed by qRT-PCR analysis in 30-paired OC tissues and corresponding adjacent non-tumor tissues at mRNA level (*n* = 30). **C** CRAT expression was analyzed by immunohistochemistry (IHC) staining assay in another 122-paired OC tissues and corresponding adjacent non-tumor tissues (*n* = 122). **D**, **E** CRAT expression was analyzed by qRT-PCR and western blot analysis in five human OC cell lines and one normal ovarian cell line. Data presented as mean ± SEM of triplicate independent experiments, *P < 0.05. **F** Kaplan–Meier survival curves were used for comparing overall survival (OS) of OC patients (*n* = 122) with different CRAT expression levels. **G**, **H** The online Kaplan–Meier plotter was used for analysis of overall survival (OS) and progression-free survival (PFS) of OC patients with different CRAT expression levels.

First, we analyzed the protein expression level of CRAT in OC with the TCGA dataset using the online UALCAN platform [10]. CRAT was markedly downregulated in OC samples as compared to normal ovarian tissues (Fig. 1A). Consistently, qRT-PCR analysis in OC tissues and corresponding adjacent non-tumor tissues (*n* = 30) revealed that the expression of CRAT was also decreased in OC at mRNA level (Fig. 1B). A significant downregulation of CRAT was further confirmed in another 122-paired OC tissues and corresponding adjacent non-tumor tissues by immunohistochemistry (IHC) analysis (Fig. 1C). To provide further support, CRAT expression was detected in five human OC cell lines and one normal ovarian cell line by qRT-PCR and western blot assays. In keeping with the results in OC tissues, CRAT expression was also dramatically lower in OC cell lines as compared with normal ovarian cell line (Fig. 1D and E). Correlation analysis between CRAT expression and clinicopathologic features of OC patients indicated that CRAT expression was negatively associated with tumor size and lymphatic metastasis in OC patients (Supplementary Table 3). Furthermore, using the median expression level of CRAT as the cut-off, OC patients with lower CRAT expression have significant shorter survival time than patients with higher CRAT expression (Fig. 1F). In agreement with this, prognostic analysis using the online Kaplan-Meier plotter database also indicated that OC patients with low CRAT expression had poorer overall survival (OS), although failed to reach statistical significance, and progression-free survival (PFS) than those with high CRAT expression (Fig. 1G and H). Together, these results indicate that the expression of CRAT is frequently downregulated in OC, which predicts poor survival of OC patients. CRAT has been reported to locate not only in mitochondrial but also in several other subcellular regions, such as cytoplasm [5]. To clarify the localization CRAT in OC cells, an immunofluorescence double-staining assay was conducted in A2780 and ES2 cells expressing relative high CRAT level. The results showed a significant co-localization of CRAT and mitochondrial in both of A2780 and ES2 cells (Supplementary Fig. 1A), suggesting that CRAT is primarily localized in mitochondrial in OC cells.

Pan-cancer analysis of The Cancer Genome Atlas (TCGA) using the online Sangerbox 3.0 (http://sangerbox.com/home.html) indicated that CRAT was also down-regulated in nine other cancer types, including Colon adenocarcinoma (COAD), Stomach and Esophageal carcinoma (STES), Uterine Corpus Endometrial Carcinoma (UCEC), Head and Neck squamous cell carcinoma (HNSC), Kidney renal clear cell carcinoma (KIRC), Thyroid carcinoma (THCA), Rectum adenocarcinoma (READ), Bladder Urothelial Carcinoma (BLCA) and Cholangiocarcinoma (CHOL) (Supplementary Fig. 1B). Among these cancers, KIRC patients with lower CRAT expression exhibited a significant shorter survival time than those with higher CRAT (Supplementary Fig. 1C). These results suggest that CRAT may serve a tumor-suppressor gene in multiple tumor types, including OC.

### Forced expression of CRAT suppressed OC growth by inducing cell cycle arrest and apoptosis

**Fig. 2:**
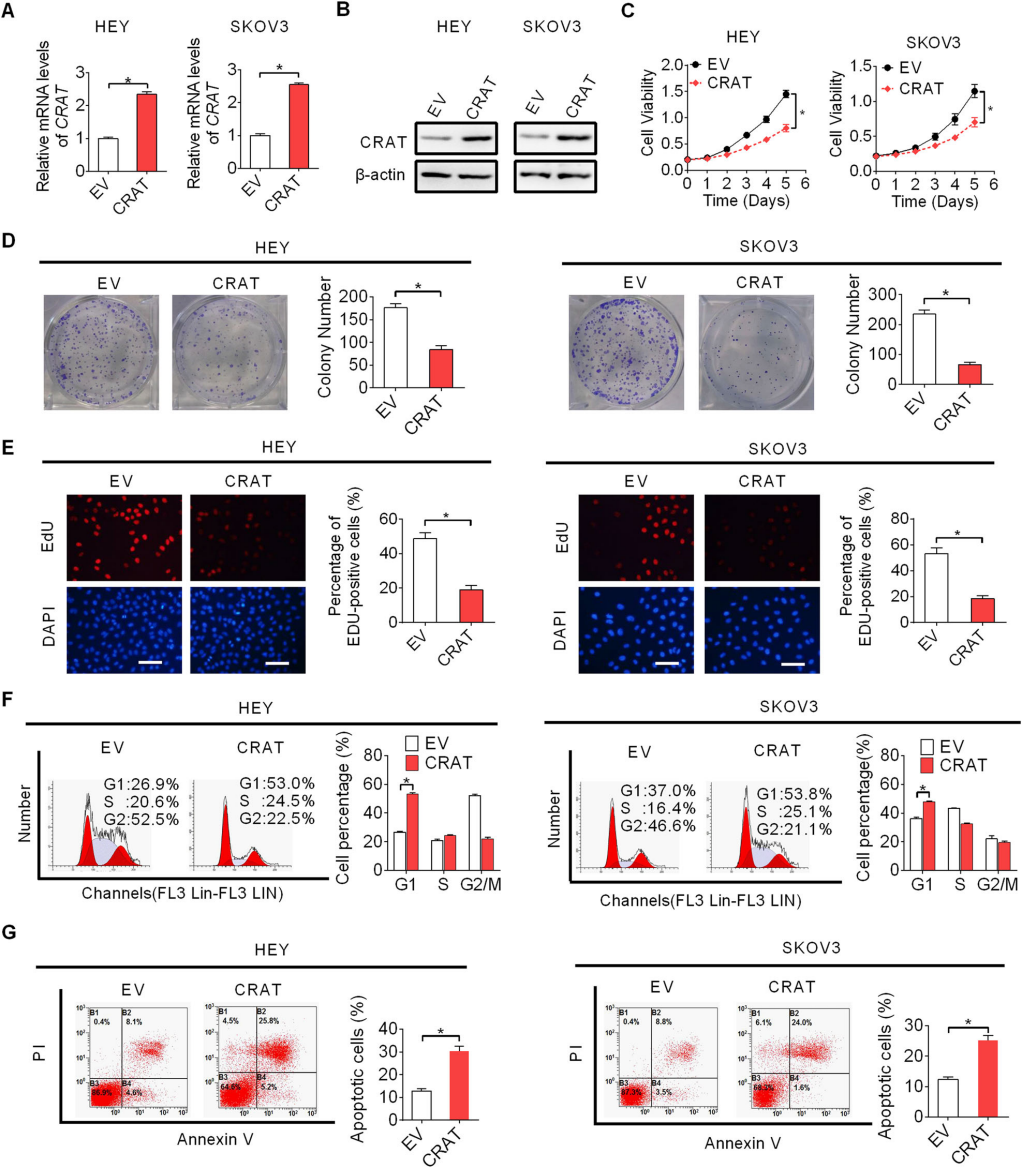
Forced expression of CRAT suppressed OC growth by inducing cell cycle arrest and apoptosis. **A**, **B** Overexpression of CRAT was determined by qRT-PCR and western blot analysis in two OC cell lines named HEY and SKOV3 (EV, empty vector; CRAT, expression vector encoding CRAT). **C**–**E** MTS cell viability **C**, colony formation **D** and EdU staining **E** assays were performed in HEY and SKOV3 cells with CRAT overexpressing. **F** The effect of CRAT overexpressing on cell cycle distribution was determined by flow cytometry analysis in HEY and SKOV3 cells. **G** The effect of CRAT overexpressing on cell apoptosis was determined by flow cytometry analysis in HEY and SKOV3 cells. Data presented as mean ± SEM of triplicate independent experiments, *P < 0.05.

We next examined the role of CRAT during OC progression. Among OC cell lines tested for CRAT expression (indicated in Fig. 1C, D), HEY and SKOV3 cells expressed relative low CRAT. We selected these two OC cell lines for CRAT overexpression by transfecting CRAT expression vector. Forced expression of CRAT (Fig. 2A, B) significantly suppressed the viability, clonogenic capacity and proliferation of HEY and SKOV3 cells (Fig. 2C–E). Mechanistically, CRAT overexpression markedly increased the proportion of OC cells in G1 phase and decreased the proportion of OC cells in S phase, indicating an induction of G1-S cell cycle arrest (Fig. 2F). Consistently, a significant positive correlation was found between CRAT and CDKN1A, also known as p21, which is a key suppressor of G1–S cell cycle transition, as revealed by the correlation analysis using the online GEPIA (Gene Expression Profiling Interactive Analysis) database (Fig. S2A). In addition, the percentage of apoptotic cells was also remarkably increased upon overexpression of CRAT in HEY and SKOV3 cells (Fig. 2G). These data suggest that CRAT suppresses OC cell proliferation in vitro, mainly via inducing cell cycle arrest and apoptosis.

### Forced expression of CRAT inhibited migration and invasion of OC cells through suppression of epithelial–mesenchymal transition (EMT)

**Fig. 3:**
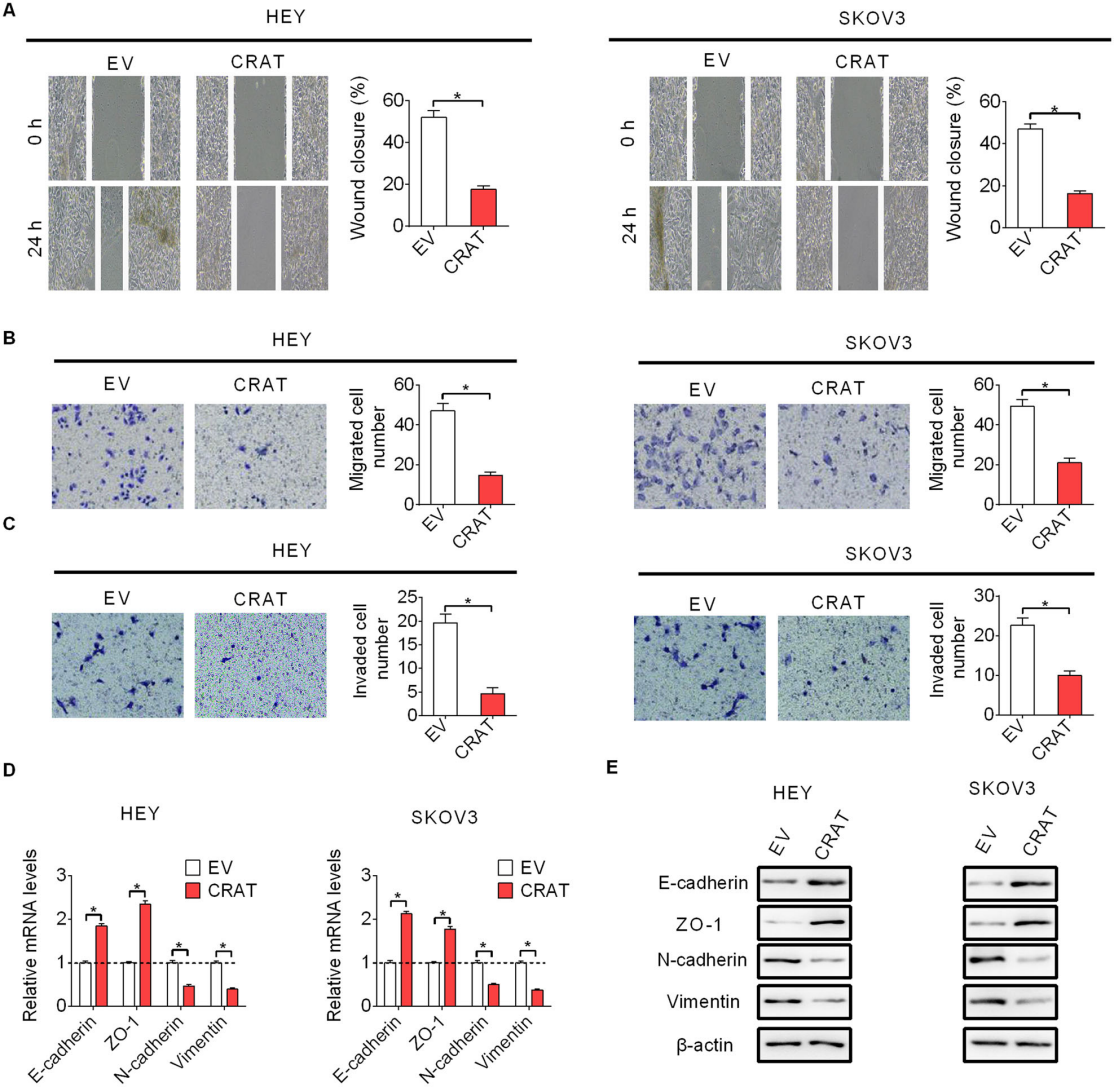
Forced expression of CRAT inhibited migration and invasion of OC cells through suppression of epithelial-mesenchymal transition (EMT). **A**, **B** The effect of CRAT overexpressing on cell migration capabilities of HEY and SKOV3 cells were evaluated by wound healing **A** and transwell migration **B** assays. **C** The effect of CRAT overexpressing on cell invasion capabilities of HEY and SKOV3 cells were evaluated by transwell invasion assays. **D**, **E** The effect of CRAT overexpressing on expressions of epithelial or mesenchymal markers was evaluated by qRT-PCR and western blot analysis in HEY and SKOV3 cells. Data presented as mean ± SEM of triplicate independent experiments, *P < 0.05.

We also examined the functional role of CRAT in migration and invasion of OC cells. Forced expression of CRAT dramatically impaired the migratory and invasive capabilities of HEY and SKOV3 cells, as indicated by wound healing and transwell migration and invasion assays (Fig. 3A–C). Mechanistically, we found that CRAT overexpression decreased the expressions of N-cadherin and Vimentin (mesenchymal cell markers), while increased the expressions of E-cadherin and ZO-1 (epithelial cell markers) in HEY and SKOV3 cells (Fig. 3D,E). In line with this, GEPIA-based correlation analysis also indicated a significant positive correlation between the mRNA expression levels of CRAT and the mRNA expression levels of E-cadherin (also known as CDH1) (Fig. S2B). Together, these results suggest that CRAT inhibits OC cell migration and invasion mainly by suppression of epithelial-mesenchymal transition (EMT) in OC cells.

### Forced expression of CRAT attenuated OC growth and metastasis in vivo

**Fig. 4:**
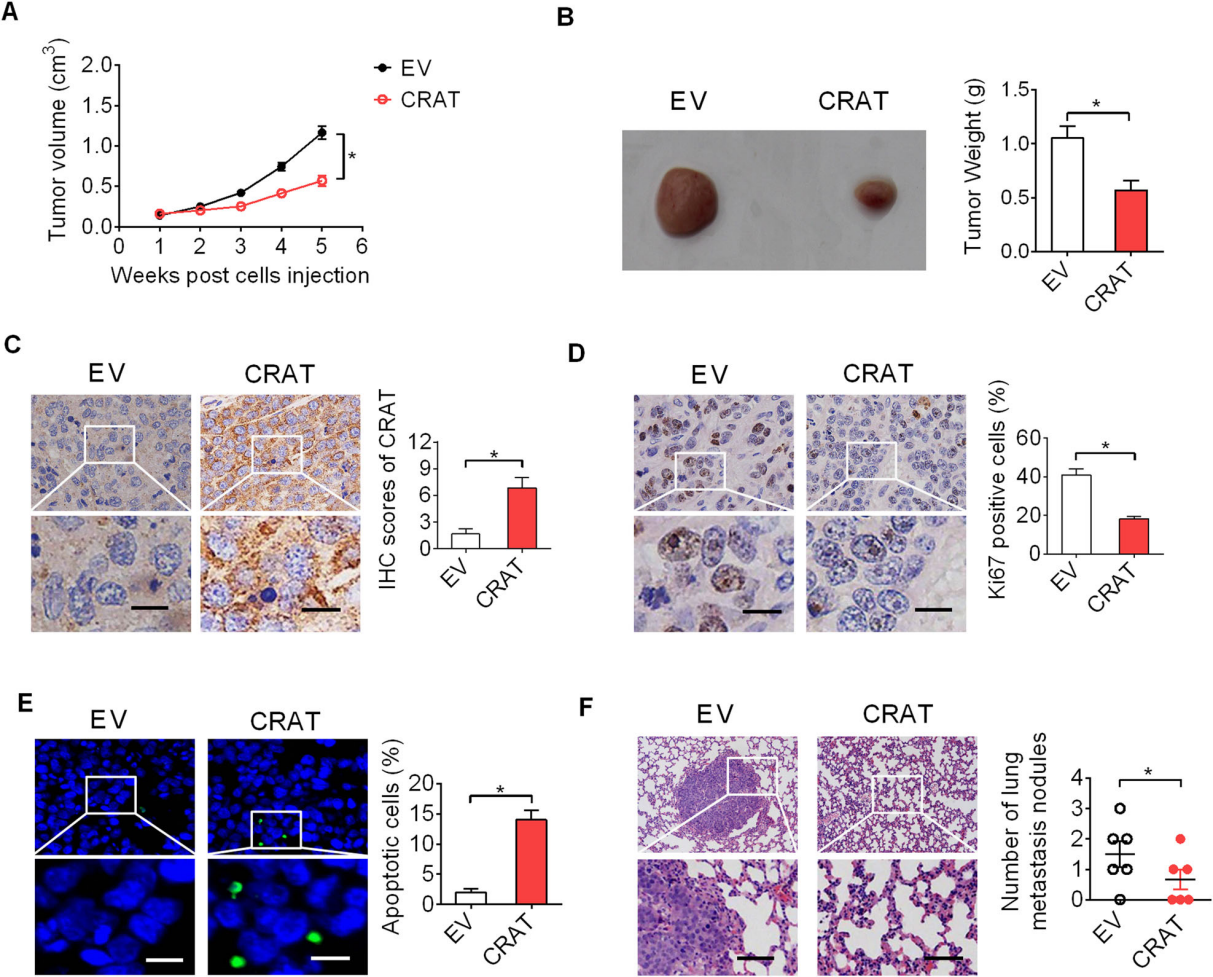
Forced expression of CRAT attenuated OC growth and metastasis in vivo. **A** Tumor growth rate of xenograft tumors (*n* = 6 mice per group) developed from CRAT stable knockdown (shCRAT) or control (shCtrl) HEY cells. **B** The weight of tumors was compared in shCRAT and shCtrl groups (*n* = 6 mice per group). **C** CRAT expression was detected by immunohistochemistry (IHC) analysis in xenograft tumors (*n* = 6 mice per group). Scale bar, 50 μm. **D** Ki-67 expression was detected by immunohistochemistry (IHC) analysis in xenograft tumors (*n* = 6 mice per group). Scale bar, 50 μm. **E** TUNEL staining assay for evaluation of cell apoptosis in xenograft tumors (*n* = 6 mice per group). Scale bars, 20 μm. **F** Lung metastases in nude mice were compared between shCRAT and shCtrl groups. Scale bars, 50 μm. (*n* = 6 mice per group). *P < 0.05.

To assess the role of CRAT in tumorigenesis of OC in vivo, stable CRAT overexpression HEY cells (Fig. S3A,S3B) were subcutaneously injected into nude mice (six mice per group) to construct xenograft model. Forced expression of CRAT significantly slowed the growth rate of HEY cells in nude mice, as determined by tumor size and weight (Fig. 4A, B). Remarkable upregulation of CRAT in xenograft tumors were validated by IHC analysis (Fig. 4C), implying that the tumor growth inhibitory role was exerted by forced expression of CRAT. In agreement with in vitro findings, fewer proliferating and more apoptotic cells were observed in xenograft tumors with CRAT overexpression, as evidenced by Ki-67 and TUNEL staining assays (Fig. 4D, E).

We also explored the effect of CRAT overexpression on OC metastasis in vivo by intravenously injecting CRAT overexpression HEY cells into the nude mice (six mice per group). The results showed that forced expression of CRAT markedly decreased the number of metastasis nodules formed in the lungs (Fig. 4F).

### CRAT knockdown promoted the growth and metastasis of OC cells

**Fig. 5:**
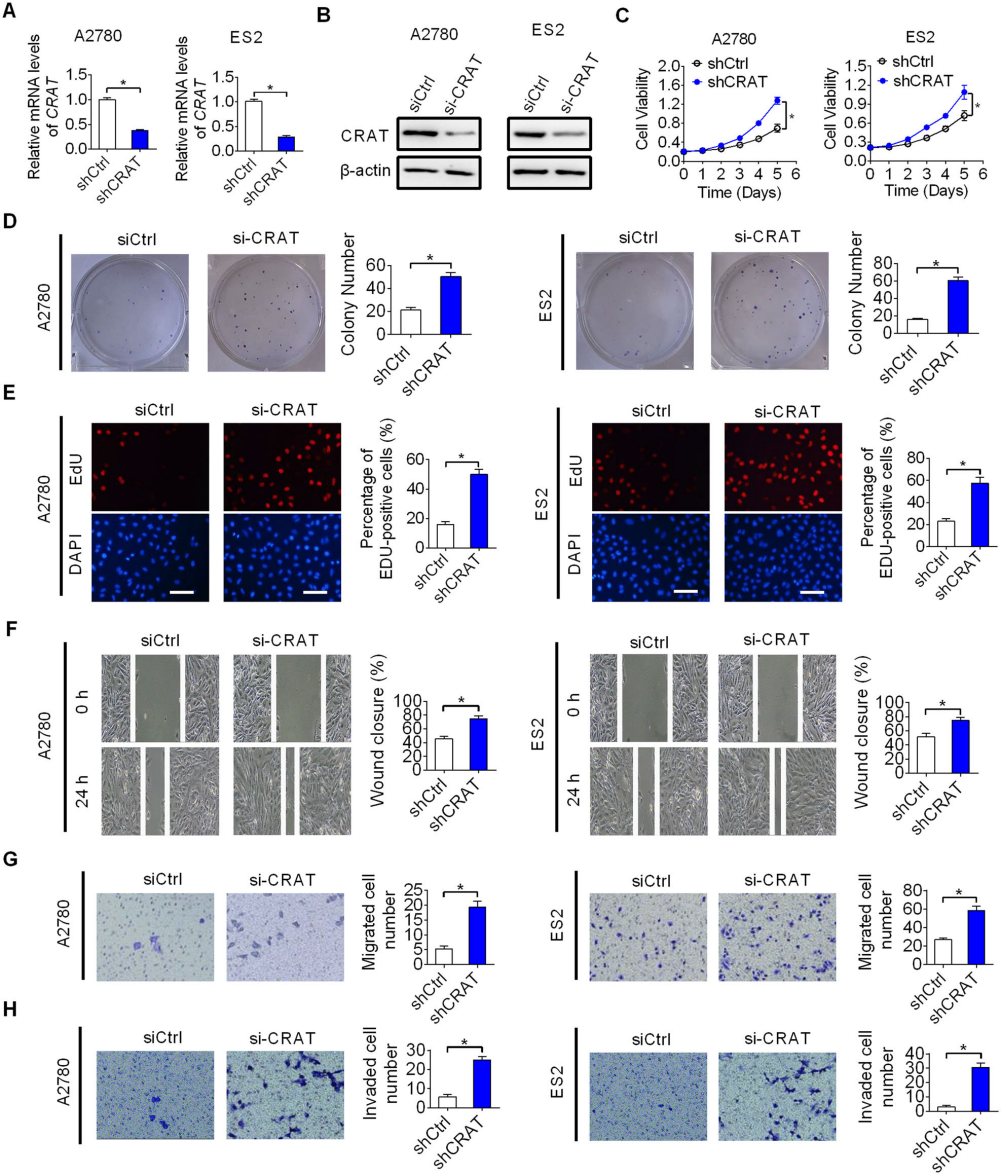
CRAT knockdown promoted the growth and metastasis of OC cells. **A**, **B** Knockdown of CRAT was determined by qRT-PCR and western blot analysis in two OC cell lines named A2780 and ES2 (siCRAT, siRNA targeting CRAT; siCtrl, control siRNA). **C**–**E** MTS cell viability **C**, colony formation **D** and EdU staining **E** assays in A2780 and ES2 cells with CRAT knocked-down. **F**, **G** Cell migration capability was evaluated by wound healing **F** and transwell migration **G** assays in A2780 and ES2 cells with CRAT knocked-down. **H** Cell invasion capability was evaluated by transwell invasion assays in A2780 and ES2 cells with CRAT knocked-down. Data presented as mean ± SEM of triplicate independent experiments, *P < 0.05.

To further confirm the role of CRAT in the inhibition of OC growth and metastasis, we knocked-down CRAT in two OC cell lines, named A2780 and ES2 with relative high CRAT expression (indicated in Fig. 1C, D). Successful knockdown of CRAT in A2780 and ES2 cells was confirmed by qRT-PCR and western blot assays (Fig. 5A, B). Knockdown of CRAT markedly increased the viability, clonogenic capacity and proliferation of A2780 and ES2 cells (Fig. 5C–E). In addition, knockdown of CRAT also remarkably promoted the migration and invasion abilities of A2780 and ES2 cells (Fig. 5F–H). These findings further support CRAT as a critical tumor suppressor in carcinogenesis and metastasis of OC.

### Downregulation of CRAT promoted mitochondrial metabolism by increasing mitochondrial biogenesis in OC cells

**Fig. 6:**
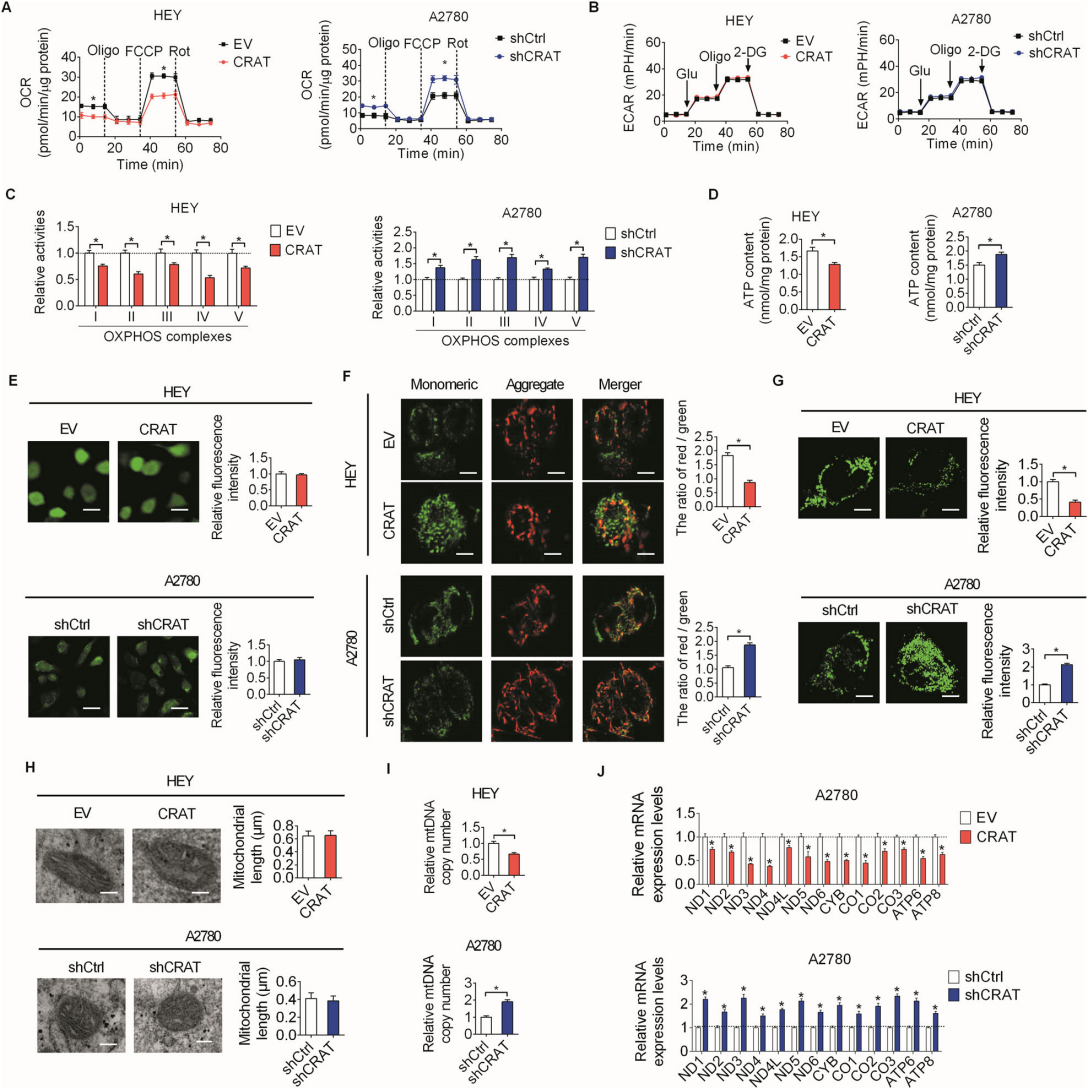
Downregulation of CRAT promoted mitochondrial metabolism by increasing mitochondrial biogenesis in OC cells. **A**, **B** The effects of CRAT overexpressing or knocking-down on oxygen consumption rate (OCR) **A** and extracellular acidification rate (ECAR) **B** were measured using a Seahorse XF96 analyzer. **C** The effects of CRAT overexpressing or knocking-down on the activities of OXPHOS complexes I–V were measured. **D**, **E** The effects of CRAT overexpressing or knocking-down on the productions of ATP and ROS were evaluated. **F** The effect of CRAT overexpressing or knocking-down on mitochondrial membrane potential (MMP) was evaluated by JC-1 staining assay. **G**, **H** The effect of CRAT overexpressing or knocking-down on the mass and length of mitochondria were determined by mitoTracker staining and TEM analysis assays. **I**, **J** The effect of CRAT overexpressing or knocking-down on the copy number of mtDNA **I** and the mRNA expression levels of mitochondrial encoded proteins that form complex I, III, IV and V (**J**) were determined by qRT-PCR analysis. Data presented as mean ± SEM of triplicate independent experiments, *P < 0.05.

Considering that CRAT is an mitochondrial-localized enzyme catalyzing the conversion between acetyl-CoA and acetylcarnitine by adding and the removing carnitine from acetyl-CoA, we therefore assessed the role of CRAT in the regulation of mitochondrial metabolism by evaluating oxygen consumption rate (OCR), extracellular acidification rate (ECAR), OXPHOS complex activity, ATP and ROS productions. The results showed that OCR, OXPHOS complex activity and ATP production were markedly decreased by CRAT overexpression in HEY cells and significantly increased by its knockdown in A2780 cells, while no notable changes in ECAR and ROS production were detected following overexpression or knockdown of CRAT (Fig. 6A–E), suggesting that CRAT knockdown OC cells preferentially utilize mitochondrial OXPHOS instead of glycolysis to confer an advantage on energy production in OC cells without increasing ROS production. In line with these findings, JC-1 staining-based determination of mitochondrial membrane potential (MMP) showed that the MMP in OC cells was significantly decreased by CRAT overexpression and increased by its knockdown (Fig. 6F). To further analyze the effect of CRAT on the mass and morphology of mitochondrial, mitoTracker staining and transmission electron microscope (TEM) analysis assays were performed in OC cells with CRAT overexpressed or knocked-down. The results showed no significant change on the length of mitochondria in OC cells when CRAT was either overexpressed or knocked-down (Fig. 6G), while the mass of mitochondrial (Fig. 6H) and copy number of mitochondrial DNA (mtDNA) (Fig. 6I) were markedly decreased when CRAT was overexpressed and increased when CRAT was knocked-down in OC cells. In line with this, the mRNA expression levels of mitochondrial encoded proteins that form complex I, III, IV and V were also markedly downregulated by CRAT overexpression and significantly upregulated by its knockdown (Fig. 6J). Together, these data indicate that CRAT plays a crucial role in the regulation of mitochondrial metabolism mainly by increasing mitochondrial biogenesis in OC cells.

### Downregulation of CRAT increased mitochondrial biogenesis by reducing the acetylation of PGC-1α

**Fig. 7:**
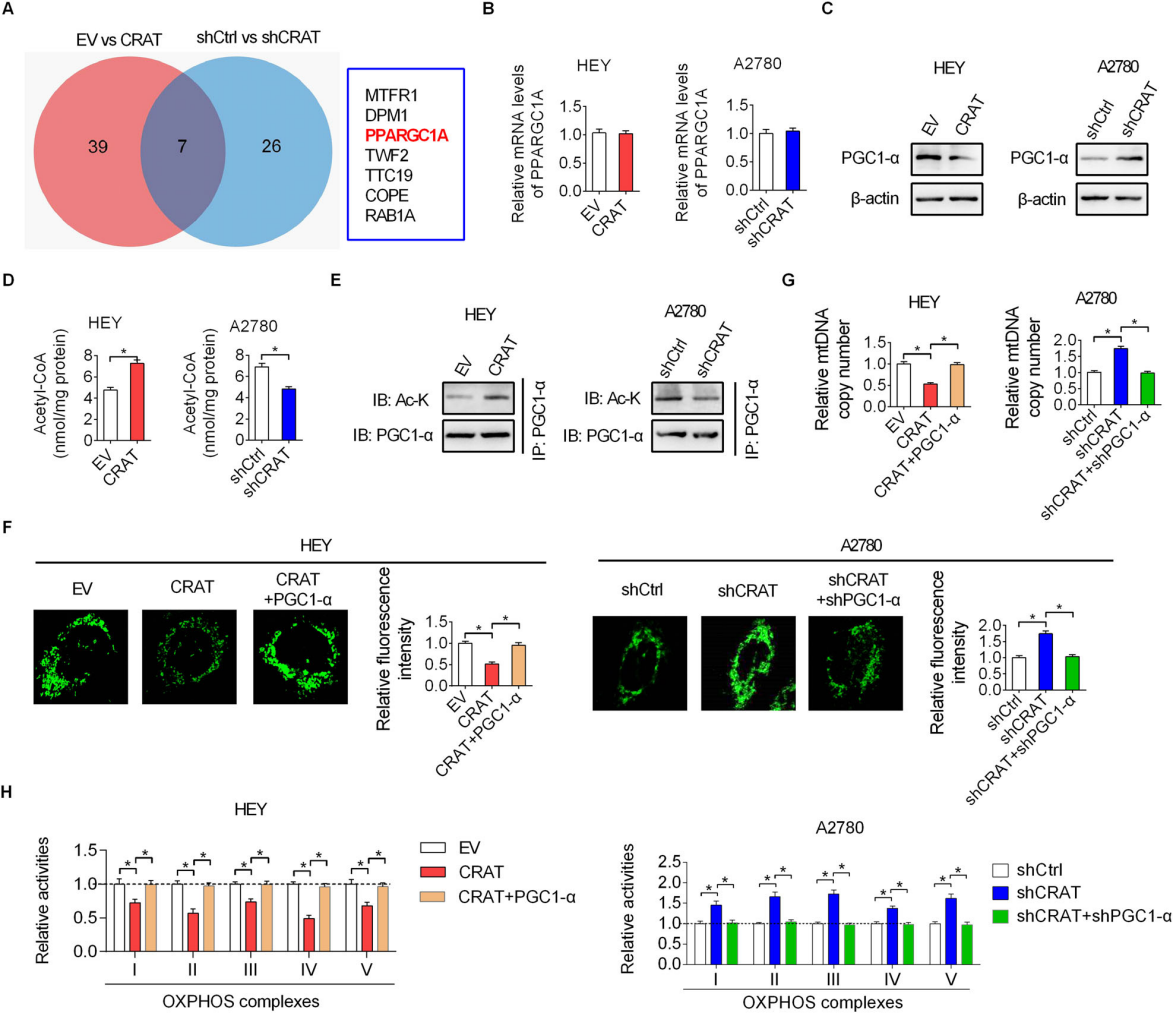
Downregulation of CRAT increased mitochondrial biogenesis by reducing the acetylation of PGC-1α. **A** The mass spectrometry (MS) assay was used to identify differently expressed proteins ( > 4-fold expression change and *p* < 0.01) between the control (EV) and CRAT overexpressing (CRAT) HEY cells, as well as between the control (shCtrl) and CRAT knocking-down (shCRAT) A2780 cells. After that, Venn diagram analysis was performed to explore the overlapping proteins that were regulated by CRAT both in HEY and A2780 cells. **B**, **C** The effect of CRAT overexpression or knockdown on the mRNA **B** and protein **C** expression levels of PGC-1α were evaluated by qRT-PCR and Western blot assays. **D** The levels of acetyl-CoA were determined in CRAT overexpression or knockdown OC cells. **E** Acetylation of PGC1-α was determined in CRAT overexpression or knockdown OC cells in the presence of MG132. **F**–**H** The mass of mitochondrial **F**, mtDNA content **G** and mRNA expression levels of mitochondrial encoded proteins **H** were determined in OC cells with indicated treatments. Data presented as mean ± SEM of triplicate independent experiments, *P < 0.05.

We then investigated the detailed mechanism underlying increased mitochondrial biogenesis caused by downregulation of CRAT in OC cells. Using the mass spectrometry (MS) assay, we found that the expression level of peroxisome proliferator-activated receptor γ coactivator 1α (PGC-1α), the master regulator of mitochondrial biogenesis and function, was significantly altered both when CRAT was overexpressed in HEY cells and knocked-down in A2780 cells (Fig. 7A). Detection of the effect of CRAT on PGC-1α expression by qRT-PCR and Western blot analysis showed that the expression of PGC-1α at protein level, but not mRNA level, was markedly decreased by CRAT overexpression and increased by CRAT knockdown (Fig. 7B, C), suggesting that PGC-1α was downregulated by CRAT at protein level. Previous studies have revealed that the protein expression level of PGC-1α was negatively regulated by acetylation [11, 12]. Given that CRAT has been recognized as a crucial regulator in the balance of mitochondrial acetyl-CoA, which is the donor of acetyl groups for protein acetylation, the effects of CRAT on the levels of acetyl-CoA and PGC-1α acetylation were thus determined. As shown in Fig. 7D, the acetyl-CoA level was obviously increased when CRAT was overexpressed in HEY cells, while decreased when CRAT was knocked-down in A2780 cells. In line with the change in total cellular acetyl-CoA level, the cytosol acetyl-CoA level was also obviously increased by CRAT overexpression and decreased by CRAT knockdown, while no notable change in mitochondrial acetyl-CoA level was observed when CRAT was either overexpressed or knocked-down, suggesting that CRAT may promote the acetylation of PGC-1α by increasing the cytoplasmic acetyl-CoA level in OC cells. Consistent with this, the acetylation of PGC-1αwas clearly increased when CRAT was overexpressed and decreased when CRAT was knocked-down (Fig. 7E). Since PGC-1α is the master mitochondrial biogenesis and function, we next asked whether downregulation of CRAT increase mitochondrial biogenesis in a PGC-1α-dependent manner. The results showed that the downregulations in the mass of mitochondrial, mtDNA content and mitochondrial encoded gene expressions caused by CRAT overexpression were significantly restored by overexpression of PGC-1α. On the contrary, those enhanced mitochondrial biogenesis phenotypes by CRAT knockdown were markedly attenuated by knockdown of PGC-1α (Fig. 7F–H). Together, above data suggest that downregulation of CRAT increased mitochondrial biogenesis by reducing acetylation of PGC-1α.

### Downregulation of CRAT promoted the growth and metastasis of OC by enhancing PGC1-α-mediated mitochondrial biogenesis

**Fig. 8:**
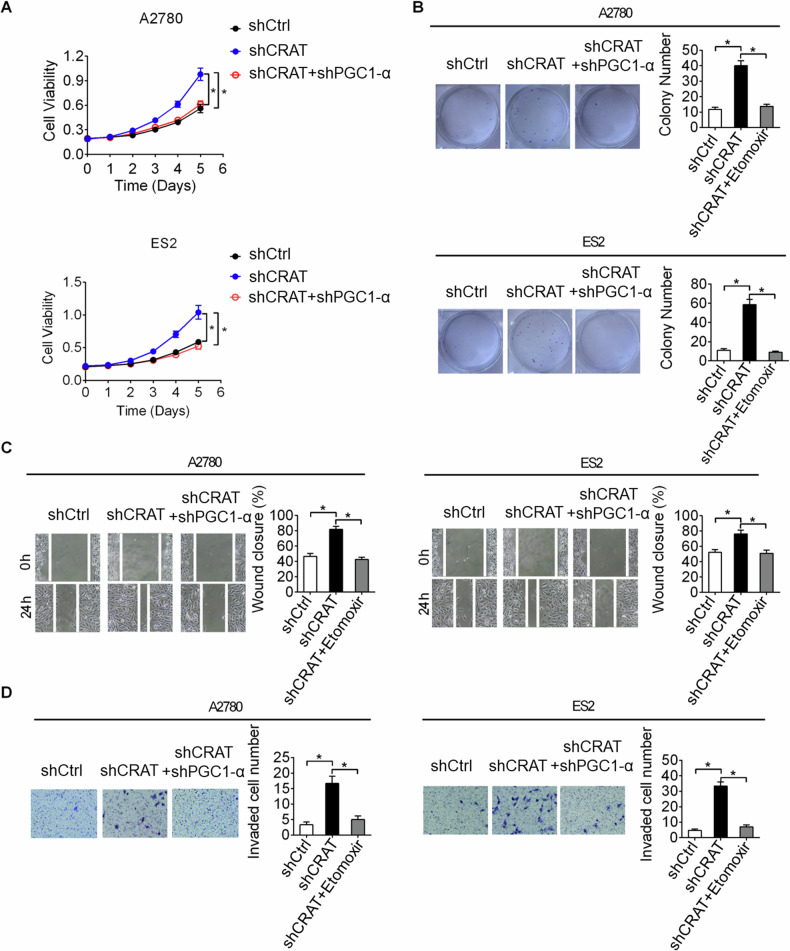
Downregulation of CRAT promoted the growth and metastasis of OC by enhancing PGC1-α-mediated mitochondrial biogenesis. **A**–**C** MTS cell proliferation **A** and colony formation **B** assays were used to evaluate the restoring effects of mitochondrial biogenesis suppression by PGC1-α silencing on CRAT downregulation-promoted OC cell proliferation and colony formation. **C**, **D** Wound healing migration **C** and transwell invasion **D** assays were used to evaluate the restoring effects of mitochondrial biogenesis suppression by PGC1-αsilencing on CRAT downregulation-promoted OC cell migration and invasion. Data presented as mean ± SEM of triplicate independent experiments, *P < 0.05.

It has been reported that enhanced mitochondrial biogenesis plays crucial roles during cancer progression [13–15]. We therefore assumed that downregulation of CRAT may promote OC progression by enhancing PGC1-α-mediated mitochondrial biogenesis. We performed MTS cell proliferation and colony formation assays, as well as wound healing migration and transwell invasion assays to evaluate the restoring effects of mitochondrial biogenesis suppression by PGC1-α knocking-down on CRAT downregulation-promoted OC progression. The results showed that suppression of mitochondrial biogenesis by PGC1-α silencing markedly attenuated the viability (Fig. 8A), colony formation (Fig. 8B), migration (Fig. 8C) and invasion (Fig. 8D) abilities of OC cells promoted by CRAT knocking-down in A2780 and ES2 cells. These findings suggest that downregulation of CRAT exerts its suppressive effect on OC growth and metastasis by enhancing PGC1-α-mediated mitochondrial biogenesis.

### Decreased CRAT expression in OC cells is mainly caused by upregulation of miR-132-5p

**Fig. 9:**
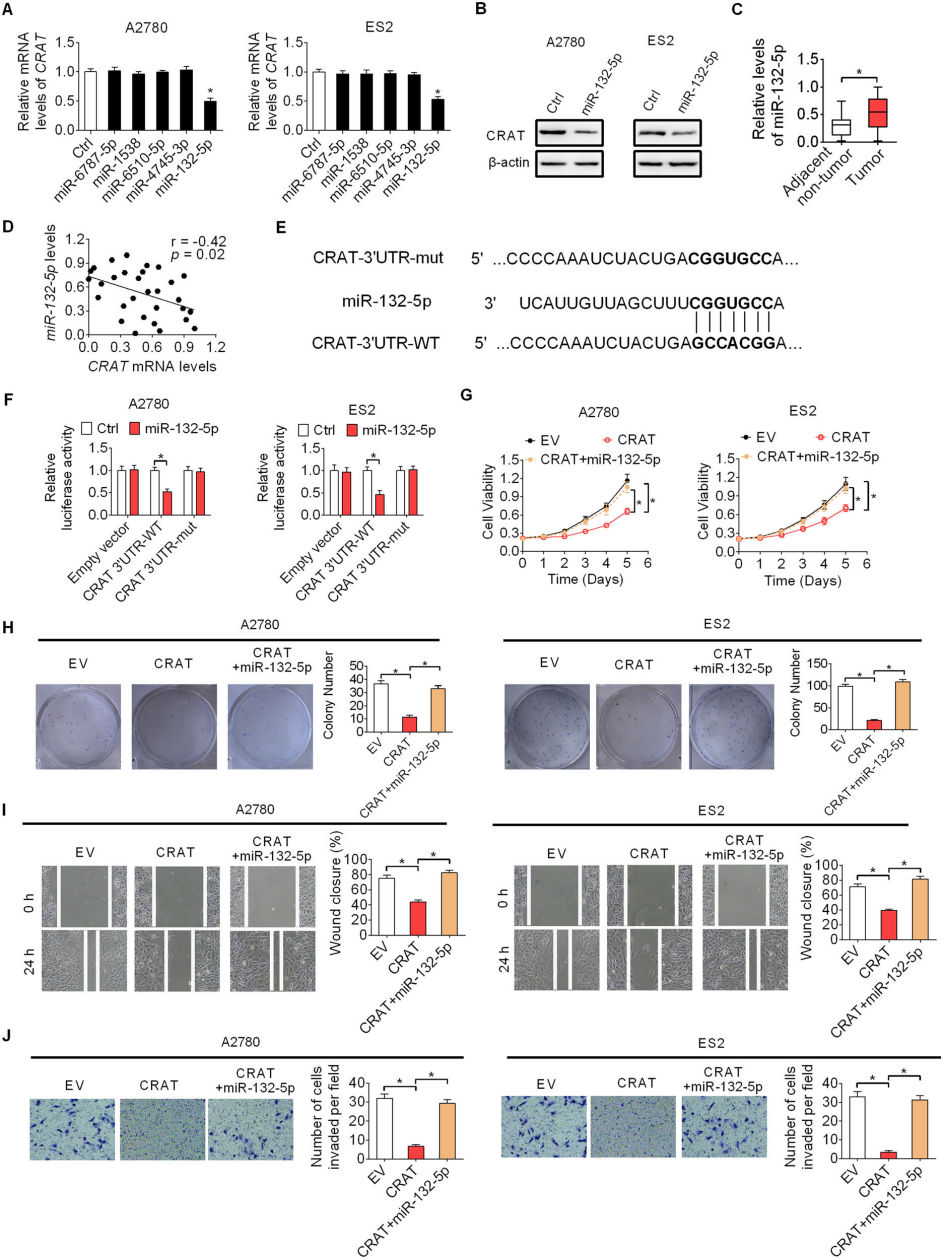
Decreased CRAT expression in OC cells is mainly caused by upregulation of miR-132-5p. **A**, **B** The mRNA and protein expressions of CRAT were determined by qRT-PCR and western blot assays in OC cells transfected with miR-132-5p. **C** miR-132-5p expression was analyzed by qRT-PCR assay in another 30-paired OC tissues and corresponding adjacent non-tumor tissues. **D** The relationship between miR-132-5p and CRAT expressions in tumor tissues from OC patients (*n* = 30). **E** Wild- and mutant-type CART 3’-UTR at the binding sites of miR-132-5p. **F** Luciferase assay was performed with CART 3’-UTR and CART 3’-UTR-mut reporters upon miR-132-5p transfection. **G**, **J** The effect of miR-132-5p transfection on CRAT downregulation-regulated in vitro OC growth **G**, **H** and metastasis **I**, **J** capabilities were evaluated. Data presented as mean ± SEM of triplicate independent experiments, *P < 0.05.

Previously studies have shown that microRNAs play critical role in gene expression regulation in human cancers [16, 17], including OC [18]. To explore potential miRNA leading to decreased CRAT expression in OC cells, a target prediction platform named microRNA Data Integration Portal (mirDIP) was utilized [19]. Among the top 5 miRNAs targeting CRAT (Fig. S5A), CRAT expression was dramatically decreased only upon miR-132-5p transfection in OC cells (Fig. 9A, B). In keeping with this, the expression levels of miR-132-5p were significantly upregulated in tumor tissues of OC as compared with their corresponding adjacent non-tumor tissues (*n* = 30) (Fig. 9C). Also, an inversely correlation was existed between the expressions of miR-132-5p and CRAT in tumor tissues from 30 OC patients (Fig. 9D). Furthermore, to confirm the binding between CRAT 3’-UTR and miR-132-5p, a luciferase reporter assay was performed with wild-type or mutated CRAT 3’-UTR coupled luciferase reporter (Fig. 9E). MiR-132-5p transfection significantly decreased the luciferase activity in OC cells with wild-type CRAT 3’-UTR upon, while not in OC cells with mutated CART 3’-UTR (Fig. 9F). Expectedly, miR-132-5p transfection markedly attenuated the in vitro proliferation and metastasis capabilities of OC cells promoted by CRAT downregulation (Fig. 9G, J). Using the online cBioPortal database, we found a significant negative association between CRAT mRNA expression and DNA methylation levels (Fig. S5B). Therefore, we do not exclude the contribution from other factors, such as DNA promoter hypermethylation, to the decreased CRAT expression in CRC cells.

Taken together, these data indicate that decreased CRAT expression in OC cells is mainly caused by upregulation of miR-132-5p.

## Discussion

Carnitine acetyltransferase (CRAT) is a critical enzyme involved in fatty acid oxidation by catalyzing both the addition and the removal of carnitine from acetyl-CoA [20]. In the present study, we demonstrate that CRAT is frequently down-regulated in OC, at least in part, due to the upregulation of miR-132-5p. Downregulation of CRAT was associated with poor survival for patients with OC, suggesting that CRAT may serve as a potential prognostic biomarker in OC. In contrast to our observations in OC, high expression of CRAT in melanoma correlates with reduced progression-free and overall survival [21]. Differences among these studies suggest the potential existence of a cancer-type-specific role for CRAT in carcinogenesis.

Frequently down-regulation of CRAT implies a potential tumor suppressive role in OC. To address this, the functional roles of CRAT in the growth of OC were firstly explored. Our results indicated that overexpression of CRAT significantly suppressed the viability and clonogenic capacity of HEY and SKOV3 cells, while knockdown of CRAT activated the viability and clonogenic capacity of A2780 and ES2 cells. Subcutaneous xenograft nude mice models further confirmed that overexpression of CRAT suppressed the in vivo growth of OC. Mechanistically, CRAT overexpression markedly induced G1-S cell cycle arrest and apoptosis of OC cells, which was supported by Ki-67 IHC and TUNEL staining assays, showing fewer proliferating and more apoptotic cells in xenograft tumors developed from CRAT overexpressing HEY cells.

We also examined the functional role of CRAT in the migration and invasion capabilities of OC cells and revealed that overexpression of CRAT led to a significant decrease of migration and invasion capabilities in HEY and SKOV3 cells, while knockdown of CRAT resulted in a clearly increase of migration and invasion capabilities in A2780 and ES2 cells. In contrast to our observations in OC, it was demonstrated that knockdown of CRAT suppressed melanoma metastasis [21], indicating a cancer-type-specific role for CRAT in cancer metastasis. Acquisition of mesenchymal phenotype is a critical feature for high metastatic cancer cells [22–24]. We found that CRAT overexpression suppressed epithelial-mesenchymal transition in HEY and SKOV3 cells, suggesting that CRAT inhibits cell migration and invasion of OC cells mainly by suppression of EMT. In concordance with the findings from in vitro migration and invasion assays, in vivo metastasis assay in nude mice also showed that overexpression of CRAT significantly increased the number of metastasis nodules formed in the lungs.

Mitochondria are important bioenergetics, biosynthetic and signaling factories that are critical for normal cell function [1]. Increasing evidence has indicated that mitochondrial metabolic alterations are closely linked to a variety processes during the development and progression human cancers [1, 2]. However, the molecular mechanisms underlying mitochondrial metabolism alterations in cancer cells remain incompletely understood. Our results showed that CRAT plays a crucial role in facilitating mitochondrial metabolism in OC cells by increasing mitochondrial biogenesis. Moreover, we revealed that downregulation of CRAT increased mitochondrial biogenesis by reducing acetyl-CoA-mediated acetylation of PGC-1α. Contrary to our findings in OC cells that downregulation of CRAT decreased acetyl-CoA level, it has been reported that CRAT deficiency induces acetyl-CoA accumulation in muscle cells, suggesting that the function of CRAT in the maintenance of acetyl-CoA balance might be disrupted in cancer cells, which still needs further investigation. As the master regulator of mitochondrial biogenesis, PGC-1α plays a central role in mitochondrial metabolism regulation [25]. It has been reported that the activity and stability of PGC-1α are mainly regulated by posttranslational modifications including phosphorylation, ubiquitination and acetylation [26]. In line with our findings in OC cells, the protein expression level of PGC-1α was also reported to be negatively regulated by acetylation [11, 12]. Actually, accumulating evidence has indicated that the role of PGC-1α in human cancers is dichotomous. The oncogenic functions of PGC-1α have been observed in multiple cancers, including prostate, breast, colon and lung cancers [13, 27–29]. By contrast, antineoplastic functions of PGC-1α were found in liver, melanomas and prostate cancers [30–32]. In ovarian cancer (OC), it was reported that suppression of PGC-1α by the aqueous extract of paris polyphylla (AEPP), a traditional Chinese medicine, could inhibit cell viability of OC cells. In line with this, we also found that down-regulation of PGC-1α by CRAT suppressed OC progression. Given that cytosolic CRAT-generated acetyl-CoA can produce malonyl-CoA, an inhibitor of fatty acid oxidation [1, 33], we speculate that, in addition to the suppression of mitochondrial biogenesis by increasing acetyl-CoA-mediated acetylation of PGC-1α, the anti-tumoral activity of CRAT may also be partially achieved by inhibiting fatty acid oxidation, which still needs further investigation.

Accumulating evidence has revealed that microRNAs play critical roles in gene expression regulation in human cancers [16]. MiR-132-5p has been reported as a suppressor of tumor metastasis in nasopharyngeal carcinoma [34]. However, the expression and functions of miR-132-5p in other human cancer types, including ovarian cancer, remain largely unclear. Here, we show that miR-132-5p is significantly upregulated and contributes to the downregulation of CRAT in OC cells. In addition, a significant negative correlation between the expressions of CRAT and miR-132-5p were also observed in tumor tissues from OC patients. In addition, luciferase reporter assay also confirmed the binding between CRAT 3’-UTR and miR-132-5p in OC cells. These data indicate that decreased CRAT expression level in OC is probably caused by elevated miR-132-5p. Moreover, our results showed that miR-132-5p markedly attenuated the in vitro growth and metastasis capabilities of OC cells promoted by CRAT downregulation. The contradictions of the function of miR-132-5p in OC and nasopharyngeal carcinoma could be explained by the fact that miR-132-5p may play distinct roles in different human cancer types. However, we cannot exclude the possibility that several other genetic and epigenetic alterations may also contribute to the downregulation of CRAT in OC cells.

Together, our study provides a molecular basis for CRAT as a critical tumor suppressor in OC progression by enhancing PGC-1α-mediated mitochondrial biogenesis and metabolism, highlighting CRAT as a promising prognostic marker and therapeutic target in the treatment of OC.

## Materials and methods

### Ovarian cancer cell lines and tissue specimens

Human ovarian cancer (OC) cell lines (A2780, ES2, HEY, OVCAR3 and SKOV3) and one ovarian cell line (IOSE80) were purchased from ATCC and cultured in DMEM medium supplemented with 10% fetal bovine serum (Sigma, USA). All cells authenticated via short tandem repeat profiling and were tested for Mycoplasma every 6 months.

A total of 152-paired OC tumor and corresponding adjacent non-tumor tissues samples were obtained from patients who underwent surgery at the First Affiliated Hospital of the Air Force Medical University. All patients have provided informed consent for obtaining their tissues specimens. The study was approved by the Human Research Ethics committee of the Air Force Medical University.

### Gene expression knocking-down and overexpressing

Gene expression was knocked-down or overexpressed in OC cells by transfecting siRNA or expression vector using lipofectamine 3000 reagent (Life Technologies) following the manufacturer’s protocols. For siRNAs, the target sequences of CRAT was 5ʹ-CCGATTTGCT

GCCAAACTCATTG-3ʹ, and the target sequences of PGC-1α was 5ʹ-ACUUAGAGGCGGA

GAAAAGGC-3ʹ.

### Real-time PCR

RNeasy Mini Kit (Qiagen, USA) was used for RNA extraction from tissues and cell lines of OC. Then, High Capacity cDNA Reverse Transcription kit (Thermo Fisher) was used for cDNA synthesis. Real-time PCR was performed using a SYBR Green PCR kit (TAKARA) with specific primers provided in the supplementary Table 2. The results were normalized to the level of β-actin. The content of mtDNA was measured by normalizing MT-ND1 gene to nuclear HGB.

### Western blot analysis

RIPA buffer was used for preparation of cell lysates. Then, equal amounts of cell lysates determined by BCA assay were resolved by SDS-PAGE. Proteins were transferred to PVDF membrane and then subjected to western blot analysis as described previously [35]. Primary antibodies used in the study were listed in supplementary Table 3.

### Immunohistochemical staining

The paraffin-embedded OC tissue section slides were deparaffinized, rehydrated and treated with 3% hydrogen peroxide. Antigen retrieval was then performed with hot citrate buffer (pH=6). After that, IHC staining was performed using an IHC detection kit (Invitrogen) as previously described [35]. Specific primary antibodies used were provided in the Supplementary Table 2. The staining results were visualized with a light Olympus microscope.

### Cell proliferation and colony formation assays

The proliferation of OC cells was analyzed by MTS cell viability assay. OC cells (1000 cells/well) were seeded onto 96-well plates and cultured over-night. After incubation with 20 μl MTS solution at 37 °C for 2 h, absorbance was detected at 490 nm with a Bio-Rad’s microplate reader.

For determination of colony formation, OC cells (500 cells/well) were seeded onto 6-well plates and cultured for two weeks. Colonies formed in each well were fixed, stained with crystal violet and numbered.

### Flow cytometry analysis for cell cycle progression and apoptosis

Cell cycle progression and apoptosis were determined with a cell cycle and Annexin V apoptosis kit (US Everbright Inc) following the manufacturer’s protocols. The results of cell cycle distribution and percentage of cell apoptosis were analyzed by flow cytometry (Beckman, Fullerton, CA).

### Xenograft tumor formation mouse model

All animal experimental procedures were performed using protocols approved by the Animal Care and Use Committee of the Air Force medical university. A total of 1× 10^6^ stable CRAT knockdown OC cells in matrigel suspension Fowere inoculated subcutaneously to 4-6-week-old female Balb/c nude mice, which were randomly assigned to two groups (six mice per group). Tumor size was measured with a caliper weekly. The mice were sacrificed at the end of the experiments. Tumors were dissected from individual mice and weighed by two blinded researchers.

### Wound healing and transwell migration and invasion assays

Wound healing assay was used for determination of cell migration ability of OC cells. OC cells were plated into 6-well plates and cultured to 95% confluency. Then, a micropipette tip was used for scratching in the middle of the wells. Detached cells were washed out by serum free cell culture medium. Scratching images were obtained with a light Olympus microscope.

Transwell migration and invasion assays were performed using a 24-well transwell chamber with matrix gel as described previously [35]. Penetrated cells were fixed with paraformaldehyde and stained with crystal violet. The numbers of migrated and invaded cells were assessed by averaging five microscopic fields per transwell membrane and triplicate independent experiments were performed.

### In vivo metastatic assay

Stable CRAT knockdown OC cells (2 × 10^6^ cells/ mice) were injected intravenously through the tail vein into the 4–6-week-old female Balb/c nude mice, which were randomly assigned to two groups (six mice per group). The mice were sacrificed at the end of the experiments and their lungs were dissected from individual mice followed by hematoxylin and eosin (H&E) staining by two blinded researchers for metastatic tumor nodules formed in the lungs.

### Oxygen consumption rate (OCR) and extracellular acidification rate (ECAR)

The OCR and ECAR of OC cells were evaluated using the XF96 Extracellular Flux Analyzer (Seahorse Bioscience) following the manufacturer’s instructions. The experiments were performed at 37 °C.

### Mitochondrial and cytosol fractionation isolation

OC cells were washed twice by ice-cold PBS and centrifuged at 1000 g at 4 °C for 2 min. The supernatant was then removed and the cytoplasmic and mitochondrial fractions of were separated using a Mitochondria/Cytosol Fractionation Kit (abcam), as per the manufacturer’s instructions.

### Measurements of mitochondrial OXPHOS complexes activities and the contents of ATP and acetyl-CoA

OC cells were trypsinized, washed with PBS and homogenized quickly before being centrifuged at 4 °C for 10 min at top speed. OXPHOS complexes activities and the contents of ATP, ROS and acetyl-CoA were then measured in the supernatants using MitoTox™ Complex I-V kits (abcam, #ab109903, #ab109904, #ab109905, #ab109906, #ab109907), ATP colorimetric/fluorometric assay kit (abcam, #ab83355) and PicoProbe Acetyl-CoA assay kit (abcam, #ab87546) according to their manufacturer’s instructions. The final results were normalized by total protein concentration.

### Evaluations of ROS content and mitochondrial membrane potential

OC cells were plated into confocal dishes and cultured overnight. After fixing in 4% paraformaldehyde for 10 min, the fluorescent probe DCFH-DA (Beyotime Biotechnology, #S0033) and JC-1 dye (Beyotime Biotechnology, #C2006) were used for evaluations of ROS and mitochondrial membrane potential as per their manufacturer’s instructions. The results were analyzed under a laser scanning confocal microscopy (Olympus).

### Mitochondrial imaging by electron microscopy and confocal microscopy

The length and mass of mitochondrial in OC cells were analyzed using transmission electron microscopy and confocal immunofluorescent microscopy with MitoTracker green FM (Molecular Probes, #M7514) as described previously [36].

### Statistical analysis

In each experiment, the sample size was chosen to ensure adequate power of detection. The variance was similar between the statistically compared groups. The data were presented as mean ± standard error of the mean (SEM). Prism 6.0 (GraphPad, San Diego, CA) software was used for data analysis. Comparisons between two groups were performed with 2-tailed unpaired student’s t-test. Comparisons among multiple groups (more than two) were performed with analysis of variance (ANOVA). Survival curves were estimated using the Kaplan-Meier method and log-rank test. *P* values < 0.05 were considered as significant.

## Supplementary information

supplementary figures and tables final version

Original Data (Full length uncropped original western blots)

## Data Availability

All data supporting the findings of this study are available from the corresponding author upon reasonable request.
