# Supplementary material for: CRAT downregulation promotes ovarian cancer progression by facilitating mitochondrial metabolism through decreasing the acetylation of PGC-1α
Source: Cell Death Discov. 2025 Jan 19;11:15. doi: 10.1038/s41420-025-02294-2 (PMC11743791; doi:10.1038/s41420-025-02294-2)
Supplement: Supplementary file 1 — supplementary figures and tables final version [file 41420_2025_2294_MOESM1_ESM.doc]

**Supplemental information**

**CRAT downregulation promotes ovarian cancer progression by facilitating mitochondrial metabolism through decreasing the acetylation of PGC-1α**

**Supplemental figures**

**Figure S1.** (A) Immunofluorescence double-staining assays of CRAT and mitochondria were performed in A2780 and ES2 cells expressing relative high CRAT level. (B) The online Sangerbox 3.0-based pan-cancer analysis of The Cancer Genome Atlas (TCGA) indicated that downregulation of CRAT exists in other nine other cancer types. (C) The online UALCAN-based Kaplan-Meier survival analysis for the prognostic significance of CRAT expression in TCGA KIRC patients.


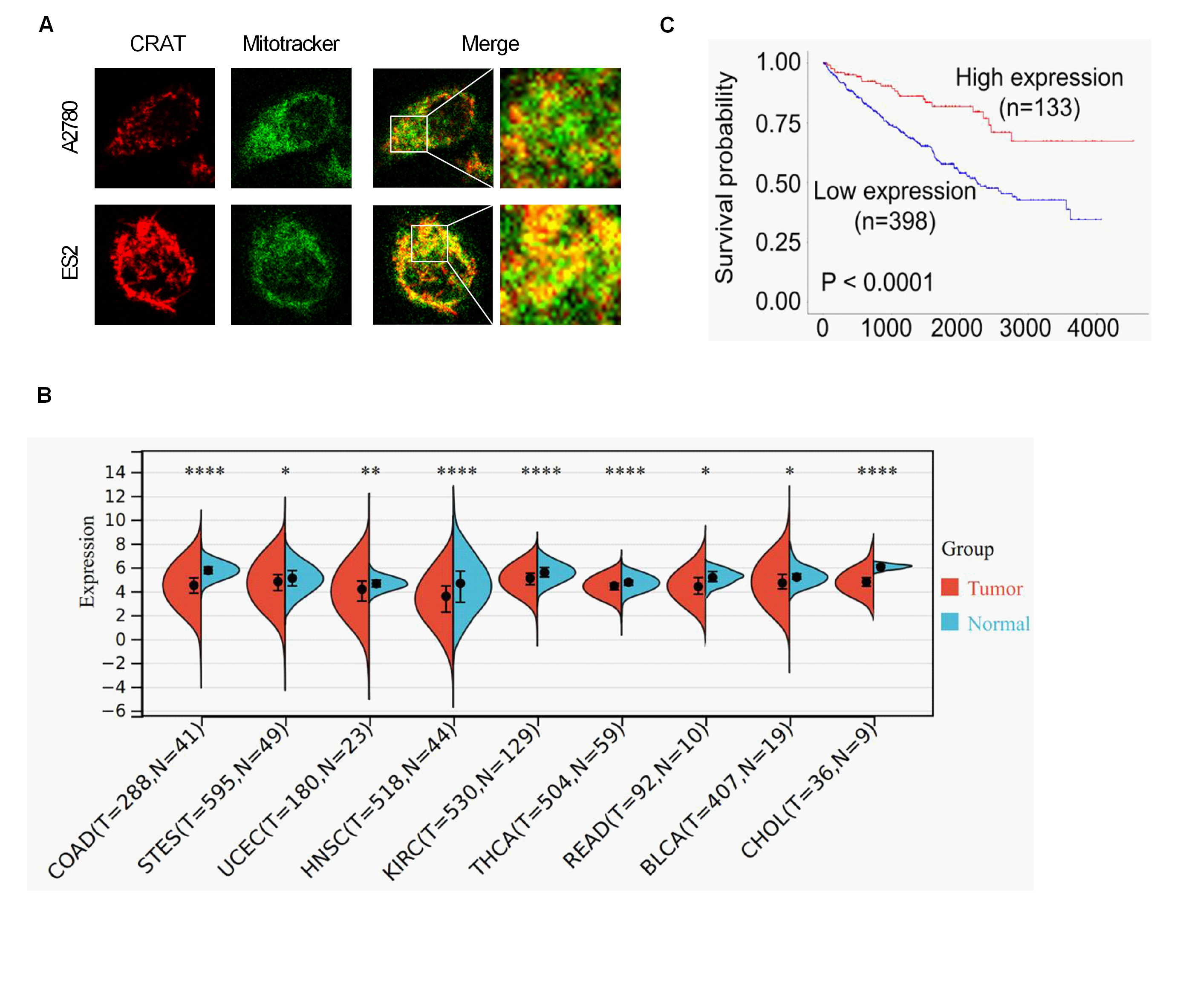


**Figure S2.** GEPIA (Gene Expression Profiling Interactive Analysis) based correlation analysis between CRAT and CDKN1A (also known as p21) and E-cadherin (also known as CDH1).


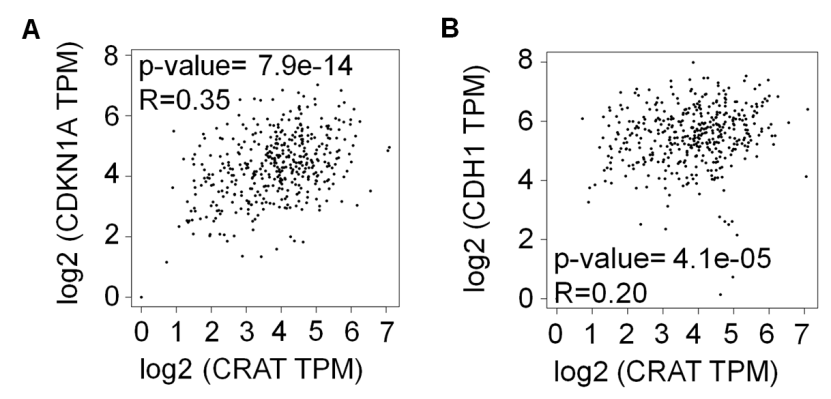


**Figure S3.** Stable overexpression of CRAT in HEY cells was determined by qRT-PCR and western blot analysis (EV, empty vector; CRAT, expression vector encoding CRAT).

**
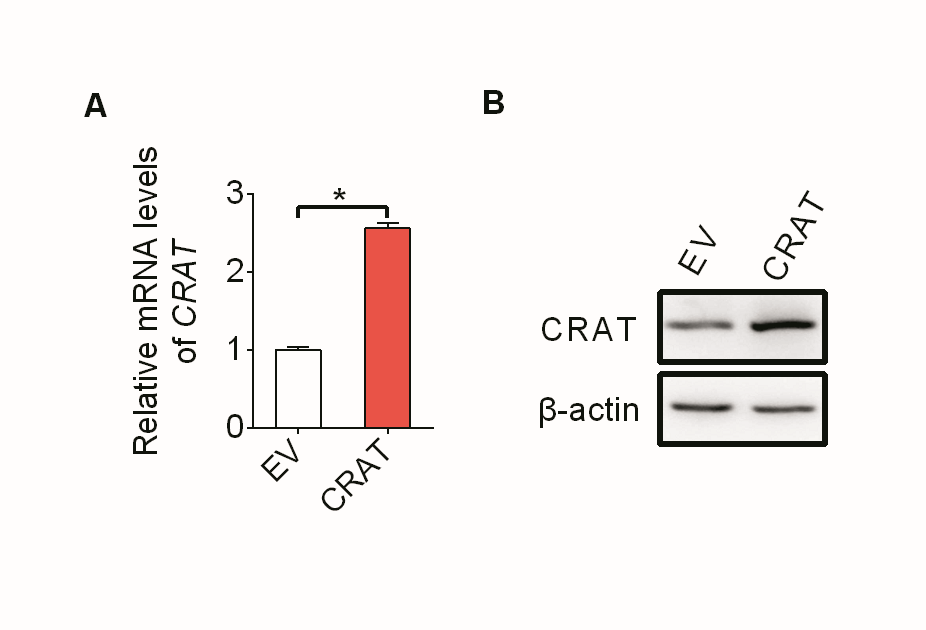
**

**Figure S4.** The levels of mitochondrial (A) and cytosol (B) acetyl-CoA were determined in CRAT overexpression or knockdown OC cells.

**
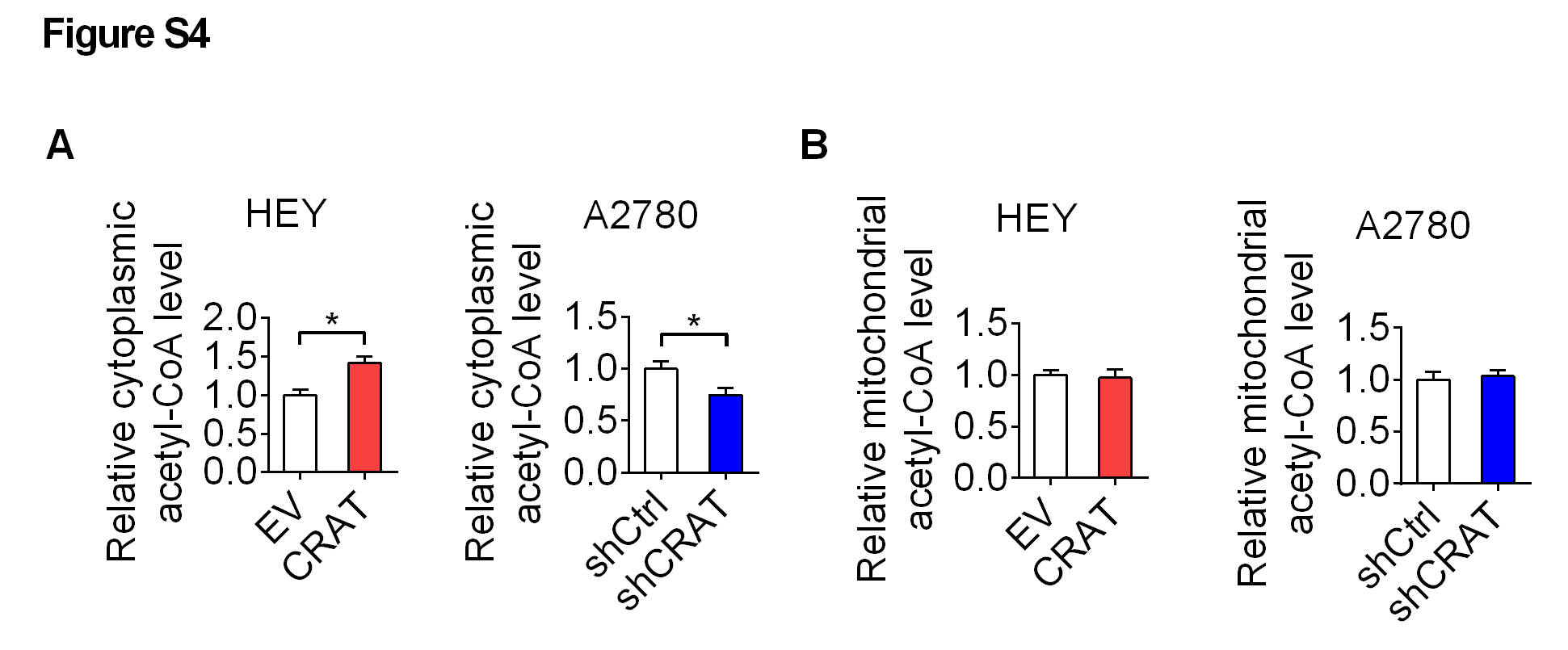
**

**Figure S5. (A)** Top five predicted miRNAs targeting CRAT by mirDIP database. **(B)** cBioPortal database-based analysis for association between CRAT mRNA expression and DNA methylation levels in CRC.


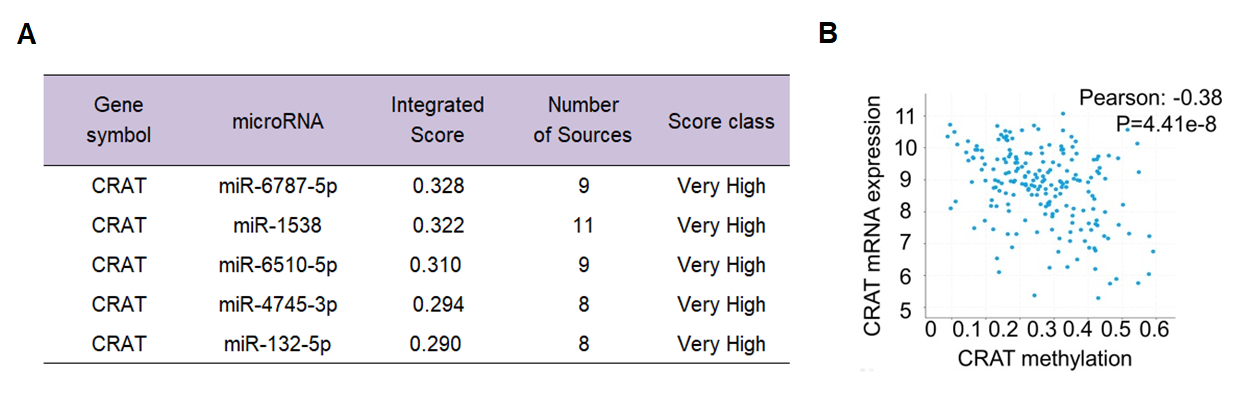


**Supplementary Tables**

**Table S1.** Sequence of primers for qRT-PCR analysis.

| **Gene** | **Forward Primer** | **Reverse Primer** |
| --- | --- | --- |
| *CRAT* | TTCACCGTGTGCCTAGATGC | CAGCGTCTTGTCGAACCAG |
| *Z0-1* | TATTATGGCACATCAGCACG | TGGGCAAACAGACCAAGC |
| *E-cadherin* | CGAGAGCTACACGTTCACGG | GGGTGTCGAGGGAAAAATAGG |
| *Vimentin* | GACGCCATCAACACCGAGTT | CTTTGTCGTTGGTTAGCTGGT |
| *N-cadherin* | TCAGGCGTCTGTAGAGGCTT | ATGCACATCCTTCGATAAGACTG |
| *β-actin* | AGGCACCAGGGCGTGAT | GCCCACATAGGAATCCTTCTGAC |
| *miR-132-5p* | ACCGTGGCTTTCGATTGTTACT | ACCTGGCATACAATGTAGATTT |
| *U6* | CTCGCTTCGGCAGCACA | AACGCTTCACGAATTTGCGT |
| *HGB* | GTGCACCTGACTCCTGAGGAGA | CCTTGATACCAACCTGCCCAG |
| *MT-ND1* | CCTCTGATTACTCCTGCCATCA | CGGCGTATTCGATGTTGAAG |
| *MT-ND2* | AGCGCTAAGCTCGCACTGAT | CTTGATGGCAGCTTCTGTGG |
| *MT-ND3* | TTACGAGTGCGGCTTCGACC | TTGTAGGGCTCATGGTAGGGGT |
| *MT-ND4* | CGGCGCAGTCATTCTCATAA | ATGCGACTGTGAGTGCGTTC |
| *MT-ND4L* | TCGCTCACACCTCATATCCTC | AAGACTAGTATGGCAATAGGCACA |
| *MT-ND5* | CCACTCTGTTCGCAGCAGTC | TGCAGGAATGCTAGGTGTGG |
| *MT-ND6* | ACAGCGATGGCTATTGAGGA | CAGCACCAATCCTACCTCCA |
| *MT-CYB* | CATCCAACATCTCCGCATGA | GGTTGAGGCGTCTGGTGAGT |
| *MT-CO1* | GTTCGCCGACCGTTGACTAT | CAGCTCGGCTCGAATAAGGA |
| *MT-CO2* | TACACCGACTACGGCGGACT | AACGTCAAGGAGTCGCAGGT |
| *MT-CO3* | TCCTAATGACCTCCGGCCTA | GTGTTACATCGCGCCATCAT |
| *MT-ATP6* | CAGGCCACCTACTCATGCAC | GGCTTGGATTAAGGCGACAG |
| *MT-ATP8* | ATACTACCGTATGGCCCACCA | GGGCTTTGGTGAGGGAGGTA |

**Table S2.** Primary antibodies used in this study.

| **Antibody** | **Company (Cat. No.)** | **Working dilutions** |
| --- | --- | --- |
| CRAT | LifeSpan (LS-C167021) | IHC, 1/200; WB, 1/800; IF:1:100 |
| ZO-1 | Proteintech (21773-1-AP) | WB, 1/1000 |
| E-cadherin | Abcam (ab1416) | WB, 1/1000 |
| Vimentin | Proteintech (10366-1-AP) | WB, 1/1000 |
| N-cadherin | Abcam (ab18203) | WB, 1/1000 |
| β-actin | Proteintech (20536-1-AP) | WB: 1/1000 |

**Table S3. Correlation between the expressions of CRAT and clinicopathologic features of OC patients (n=122).**

| Variables | No. of cases (%) | CRAT expression | | *P* value |
| --- | --- | --- | --- | --- |
|  |  | Low | High |  |
| All | 122 (100%) | 61 | 61 |  |
| Age |  |  |  |  |
| <55 | 83 (68.0%) | 44 | 39 | 0.668 |
| >=55 | 39 (32.0%) | 17 | 22 |  |
| Stage |  |  |  |  |
| I | 68 (55.7%) | 31 | 37 | 0.362 |
| II+III | 54 (44.3%) | 30 | 24 |  |
| Tumor size (cm) |  |  |  |  |
| <8 | 51 (41.8%) | 19 | 32 | **0.027** |
| >=8 | 71 (58.2% ) | 42 | 29 |  |
| Lymphatic invasion |  |  |  |  |
| No | 99 (81.1%) | 44 | 55 | **0.019** |
| Yes | 23 ( 18.9%) | 17 | 6 |  |
| Distant metastasis |  |  |  |  |
| No | 101 (82.8%) | 47 | 54 | 0.149 |
| Yes | 21 (17.2%) | 14 | 7 |  |
