# Supplementary material for: CRAT downregulation promotes ovarian cancer progression by facilitating mitochondrial metabolism through decreasing the acetylation of PGC-1α
Source: Cell Death Discov. 2025 Jan 19;11:15. doi: 10.1038/s41420-025-02294-2 (PMC11743791; doi:10.1038/s41420-025-02294-2)
Supplement: Supplementary file 2 — Original Data (Full length uncropped original western blots) [file 41420_2025_2294_MOESM2_ESM.pdf]

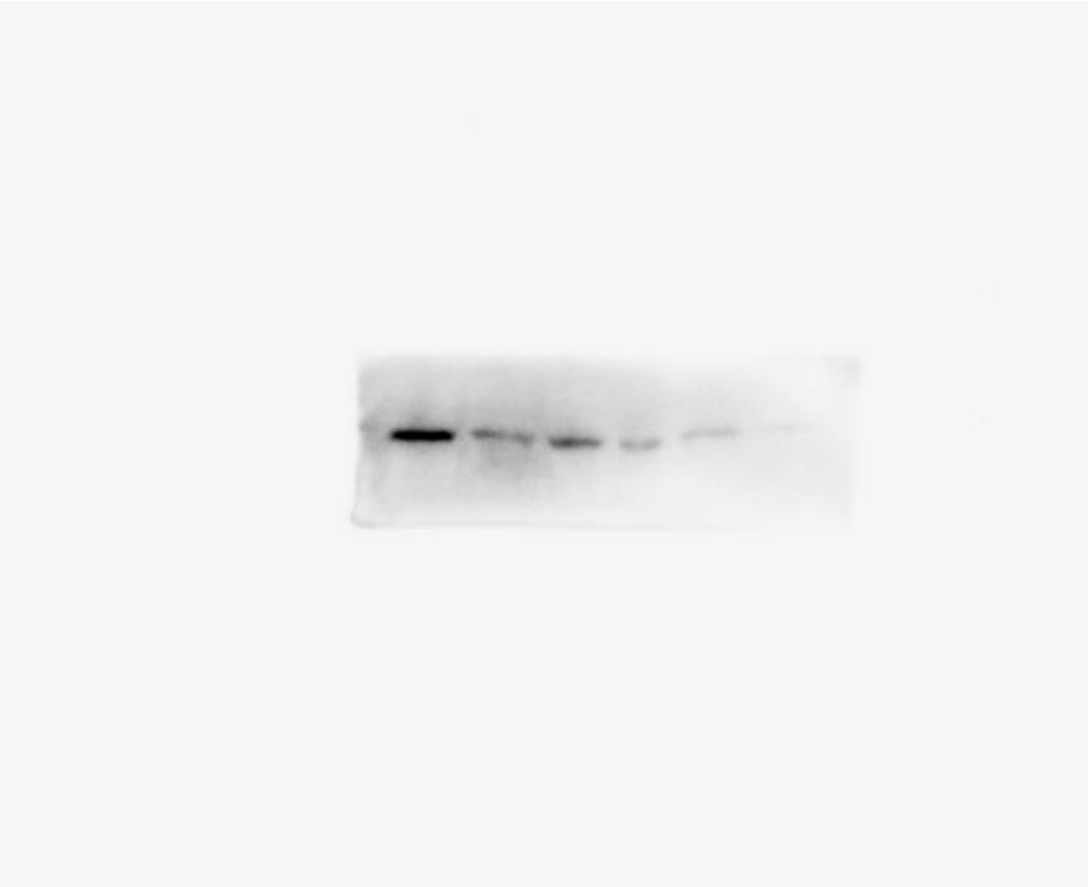

Fig 1E. IB: CRAT (sample: IOSE80, A2780, ES2, HEY, OVCAR3, SKOV3)

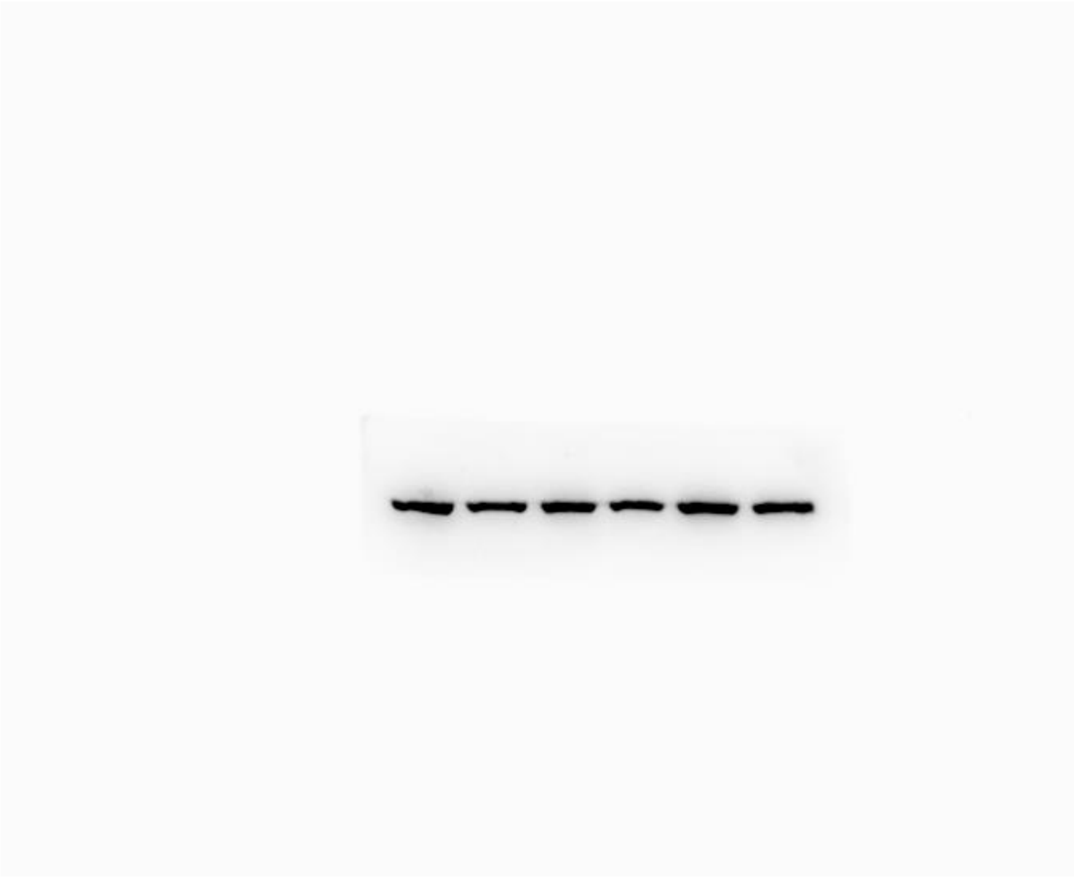

Fig 1E. IB: β-actin (sample: IOSE80, A2780, ES2, HEY, OVCAR3, SKOV3)

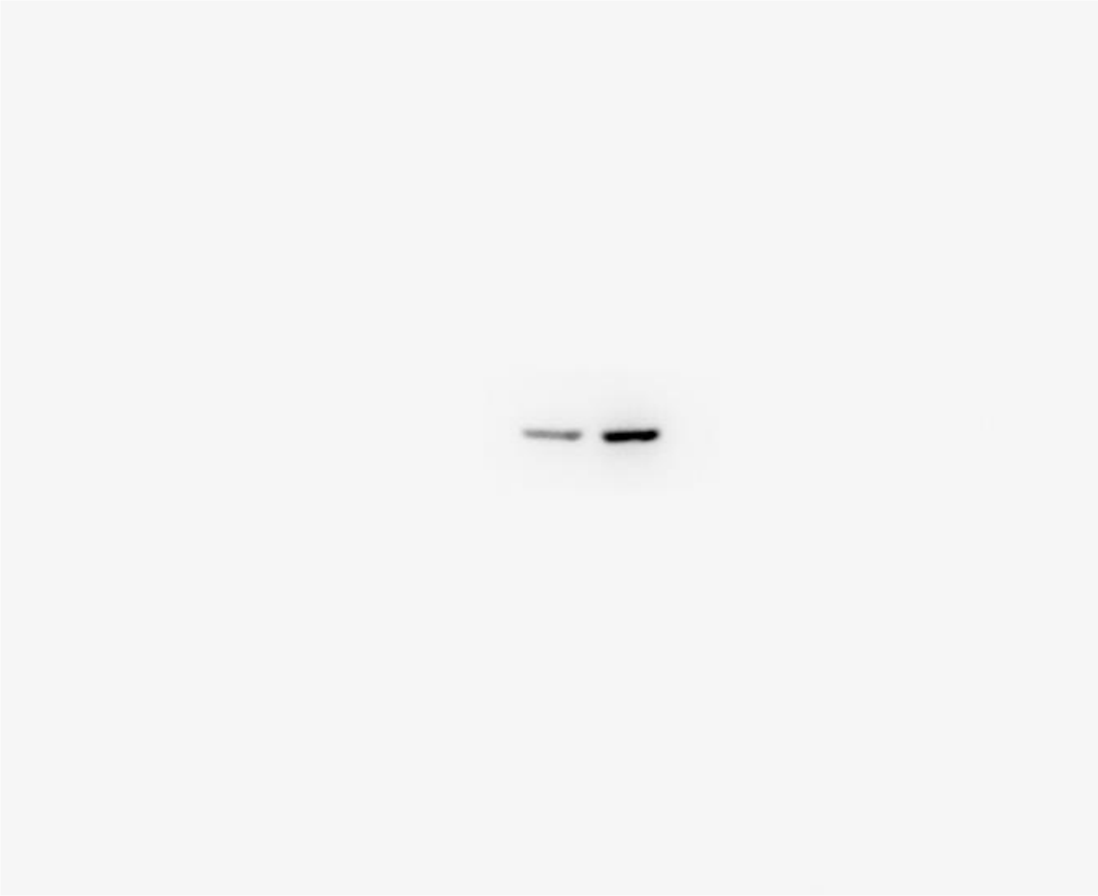

Fig 2B. IB: CRAT (HEY cell sample: EV, CRAT)

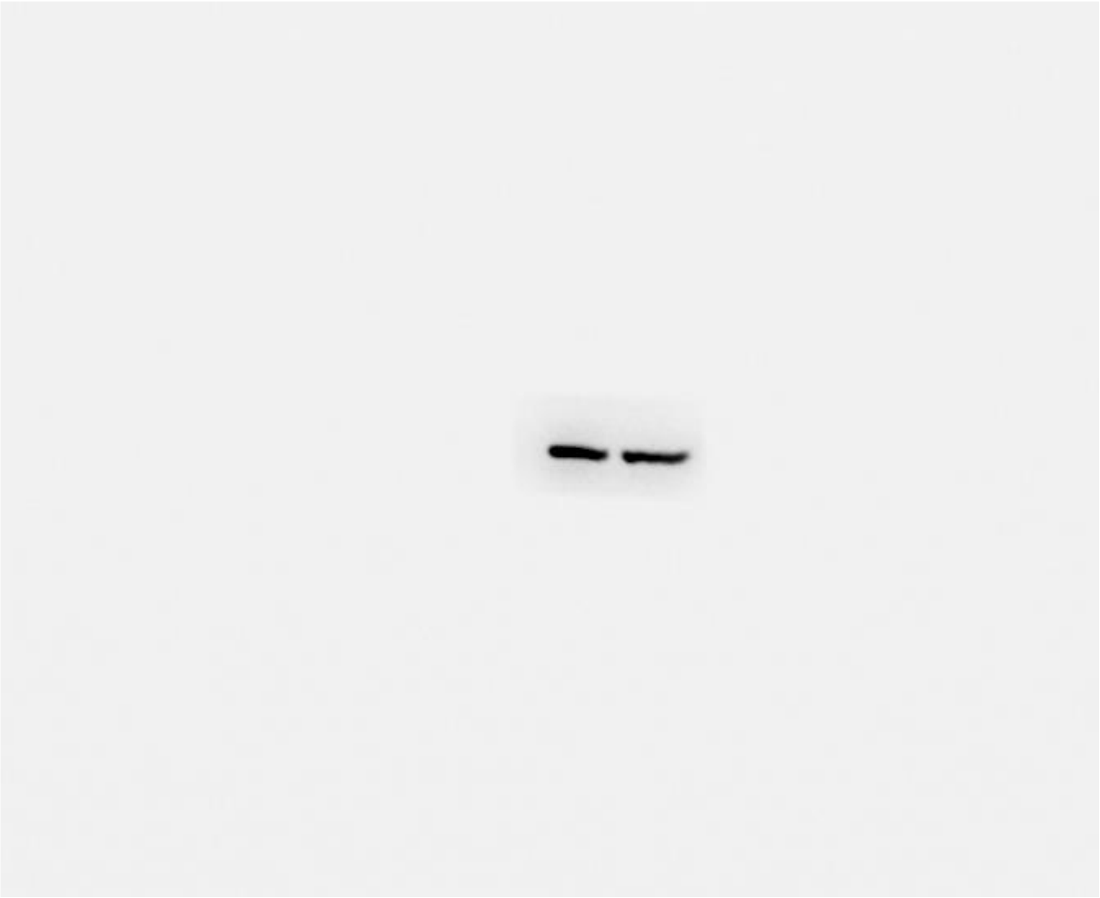

Fig 2B. IB: β-actin (HEY cell sample: EV, CRAT)

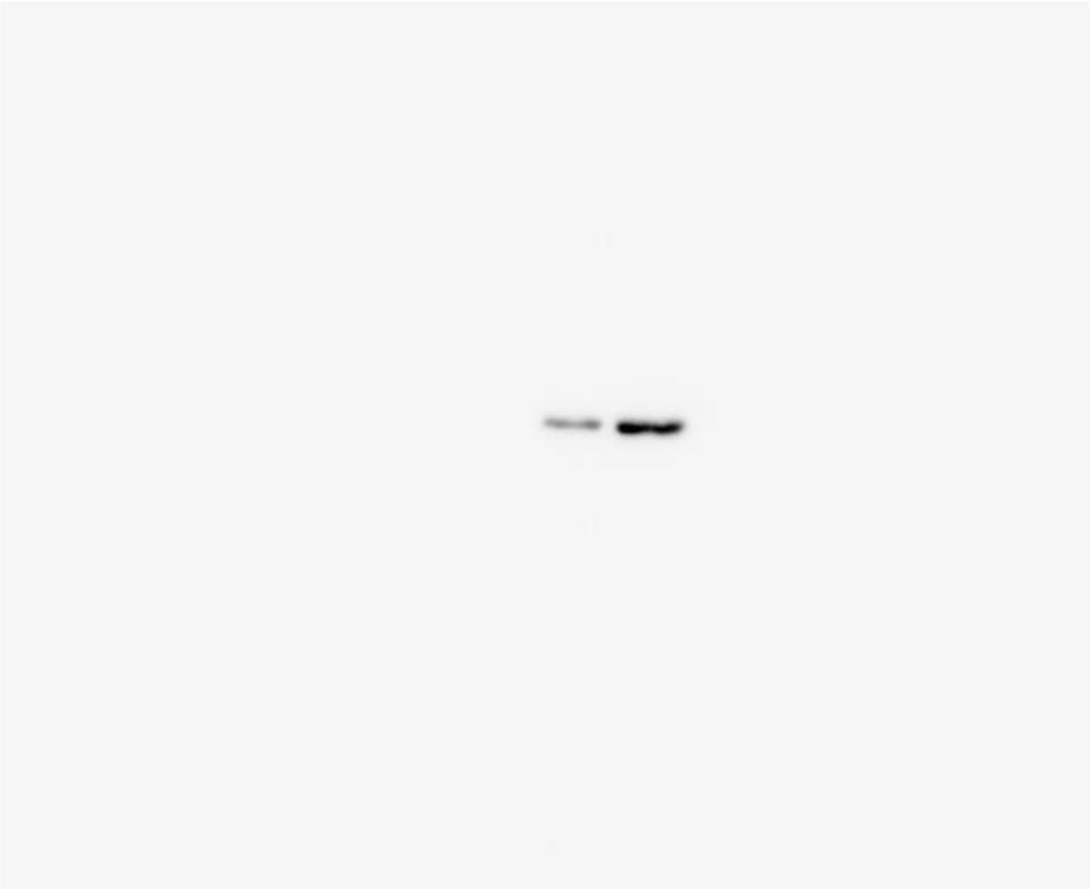

Fig 2B. IB: CRAT (SKOV3 cell sample: EV, CRAT)

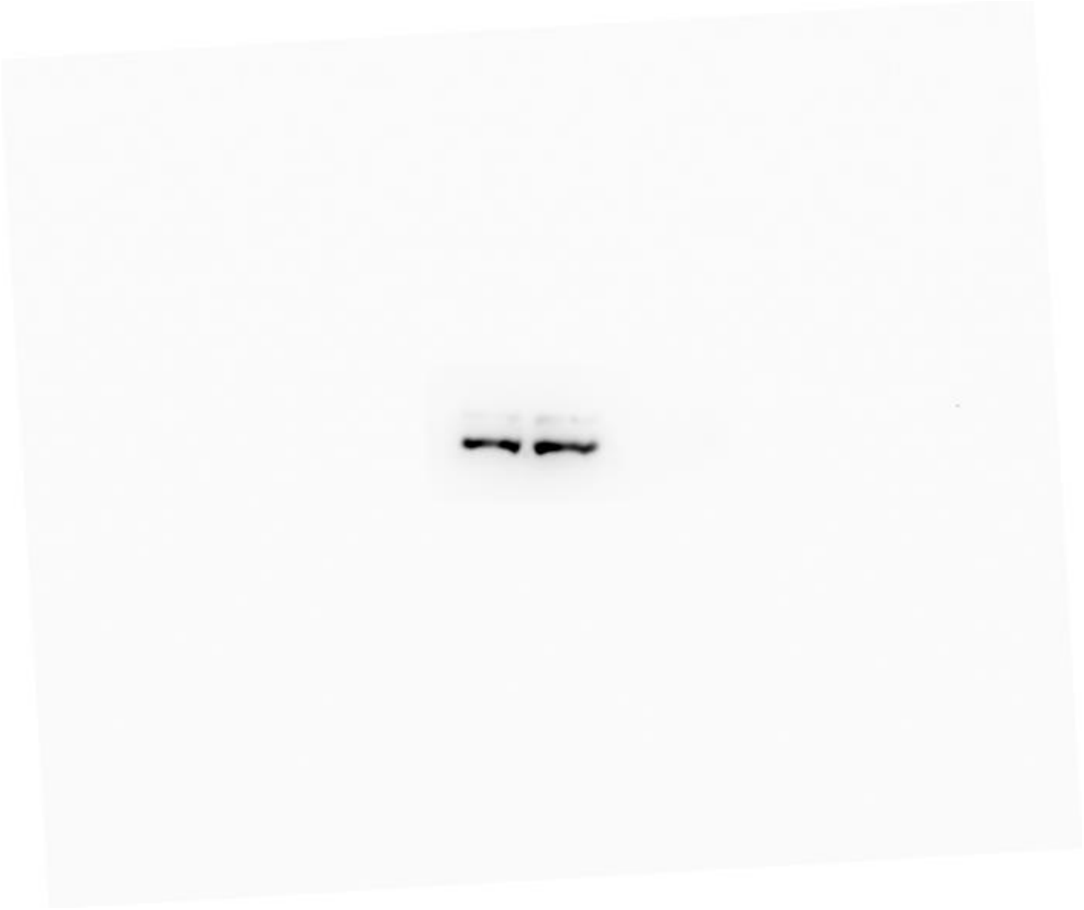

Fig 2B. IB: β-actin (SKOV3 cell sample: EV, CRAT)

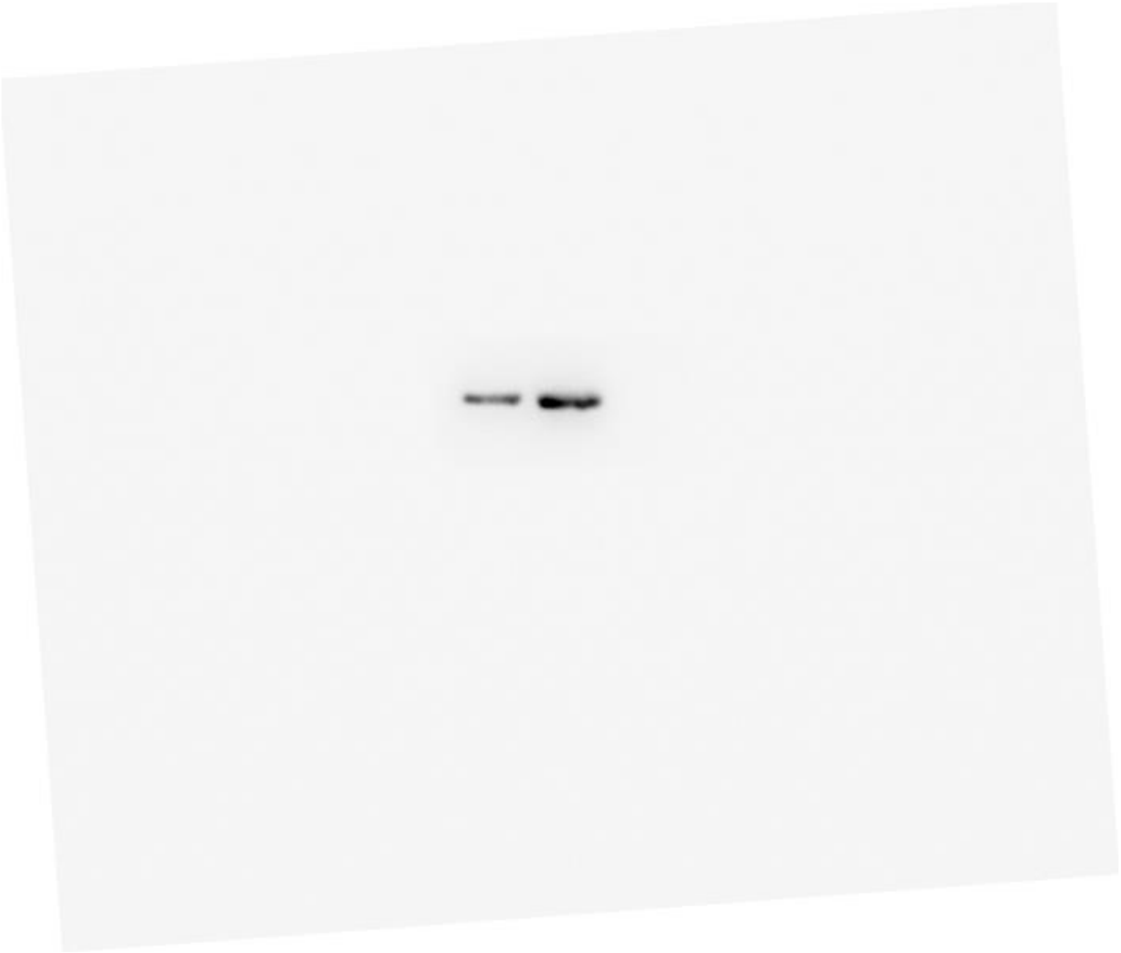

Fig 3E. IB: E-cadherin (HEY cell sample: EV, CRAT)

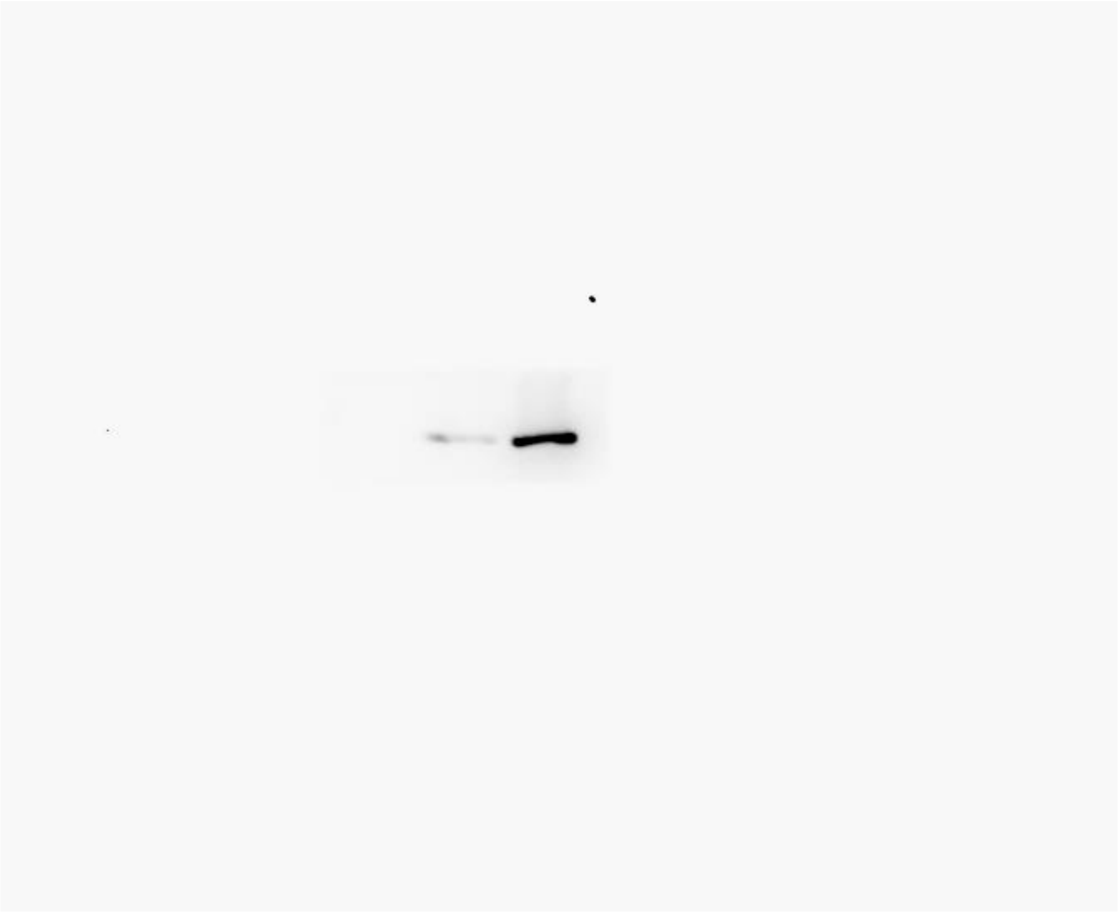

Fig 3E. IB: ZO-1 (HEY cell sample: EV, CRAT)

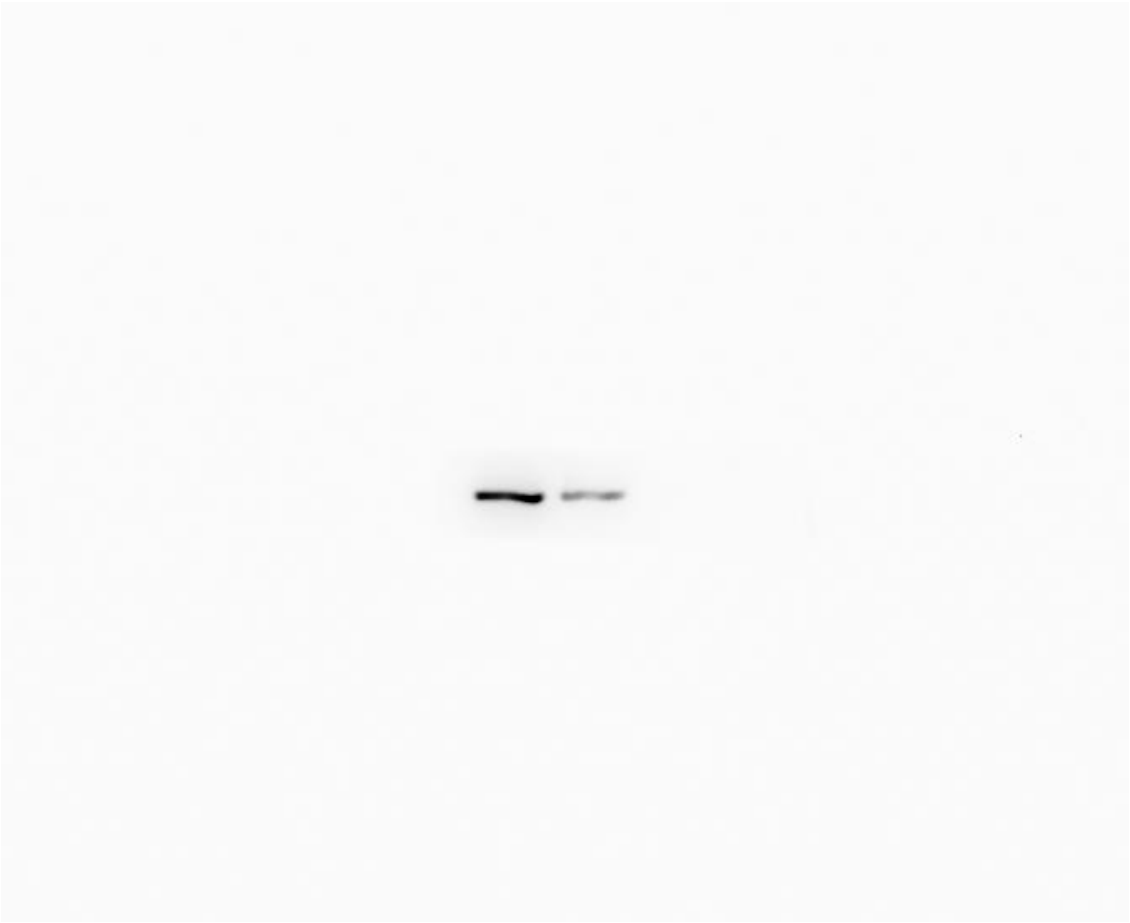

Fig 3E. IB: N-cadherin (HEY cell sample: EV, CRAT)

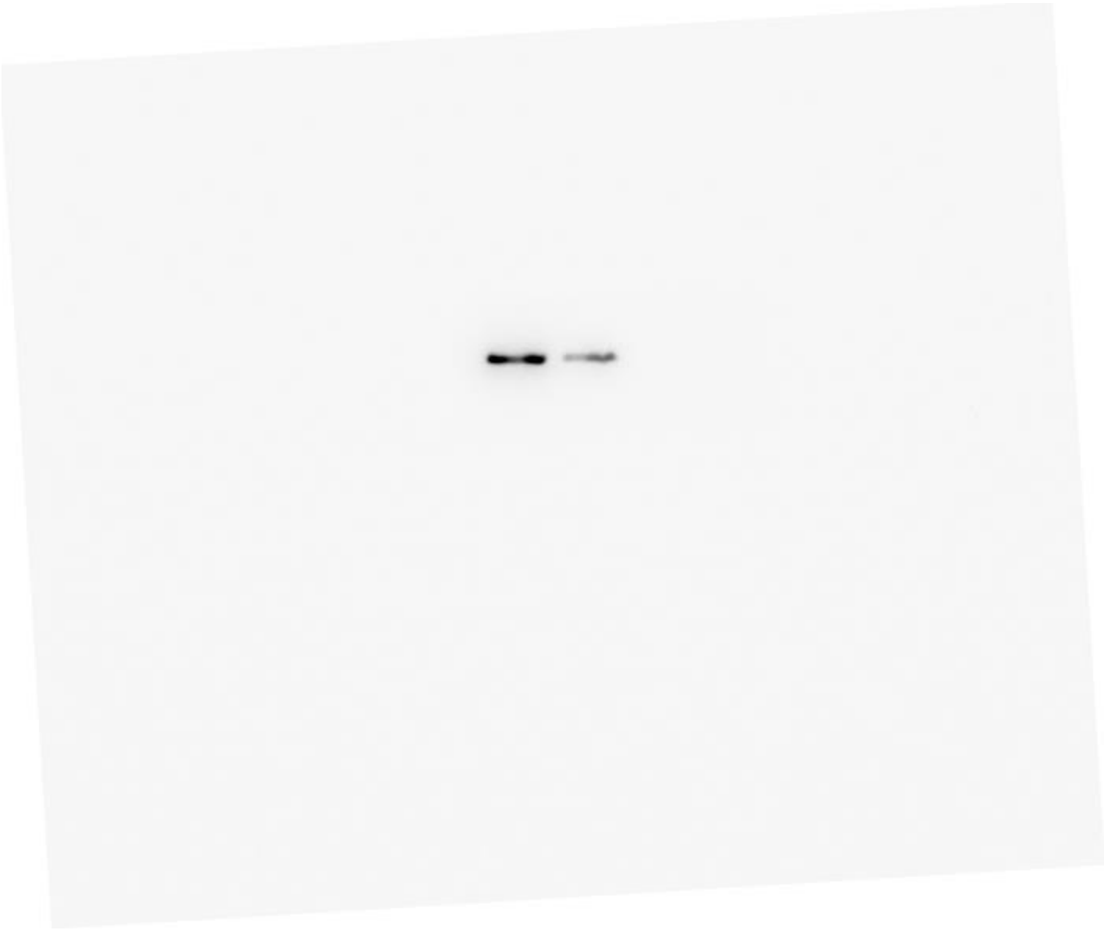

Fig 3E. IB: Vimentin (HEY cell sample: EV, CRAT)

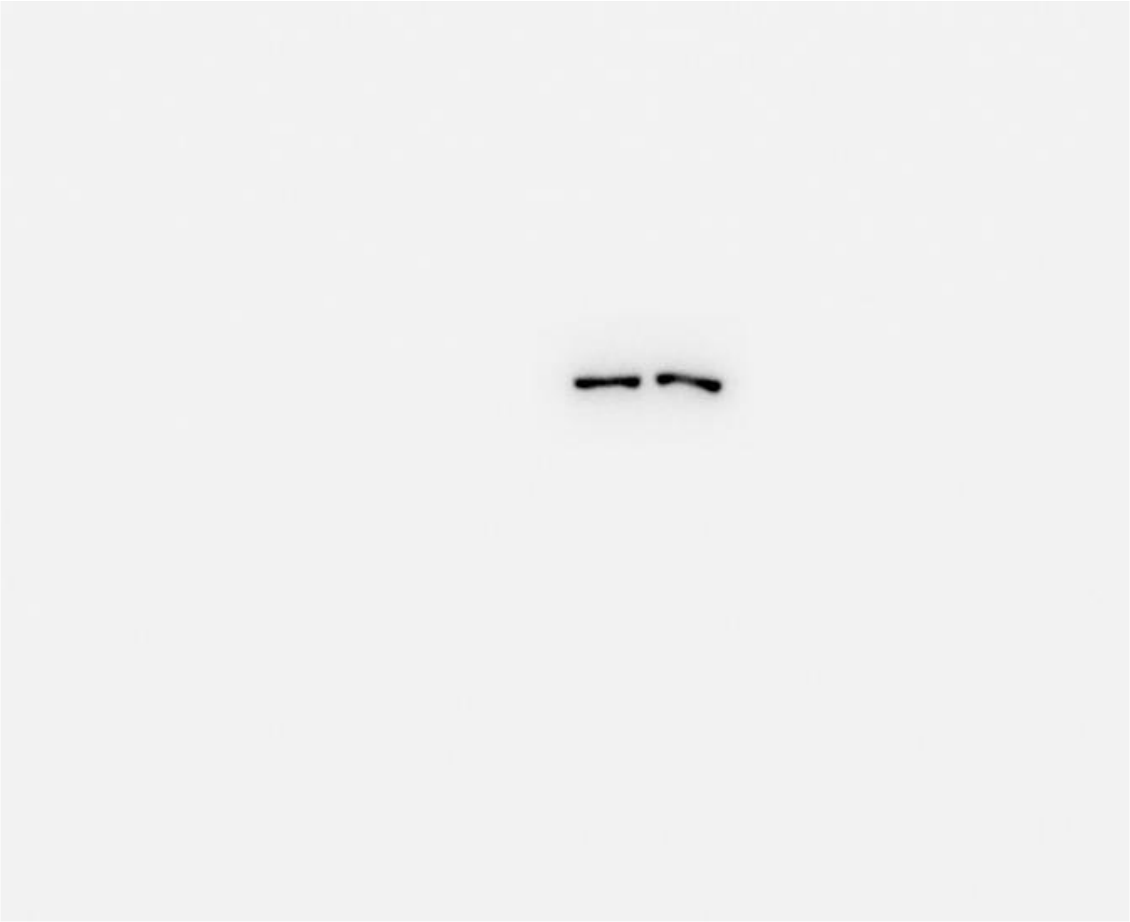

Fig 3E. IB: β-actin (HEY cell sample: EV, CRAT)

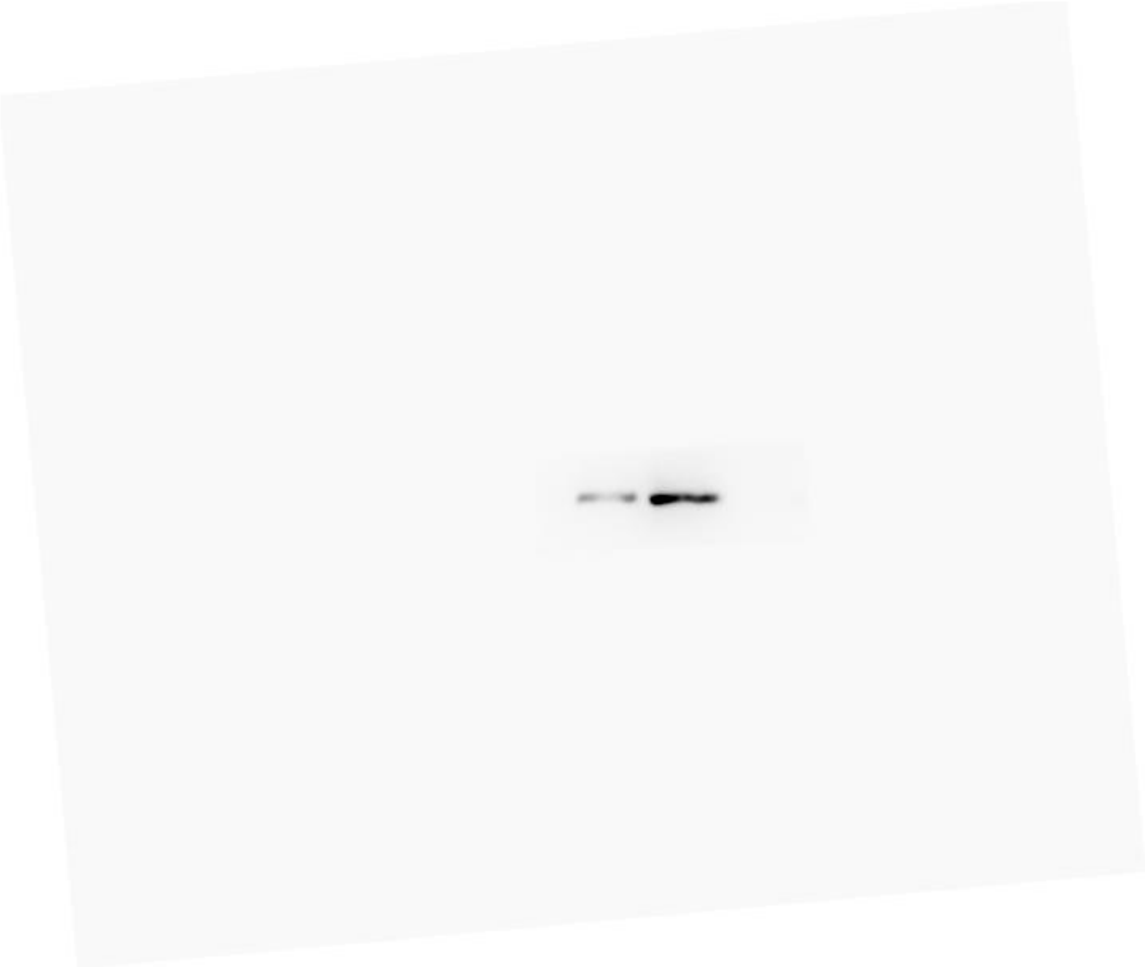

Fig 3E. IB: E-cadherin (SKOV3 cell sample: EV, CRAT)

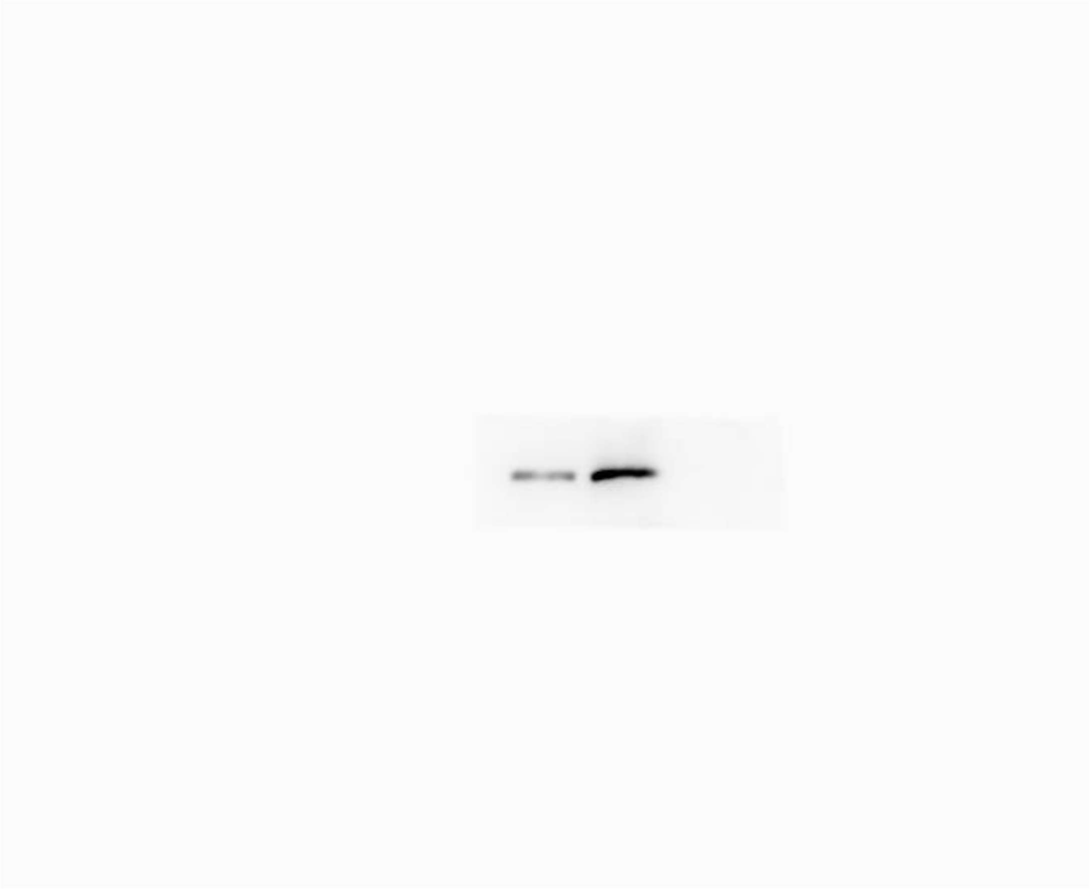

Fig 3E. IB: ZO-1 (SKOV3 cell sample: EV, CRAT)

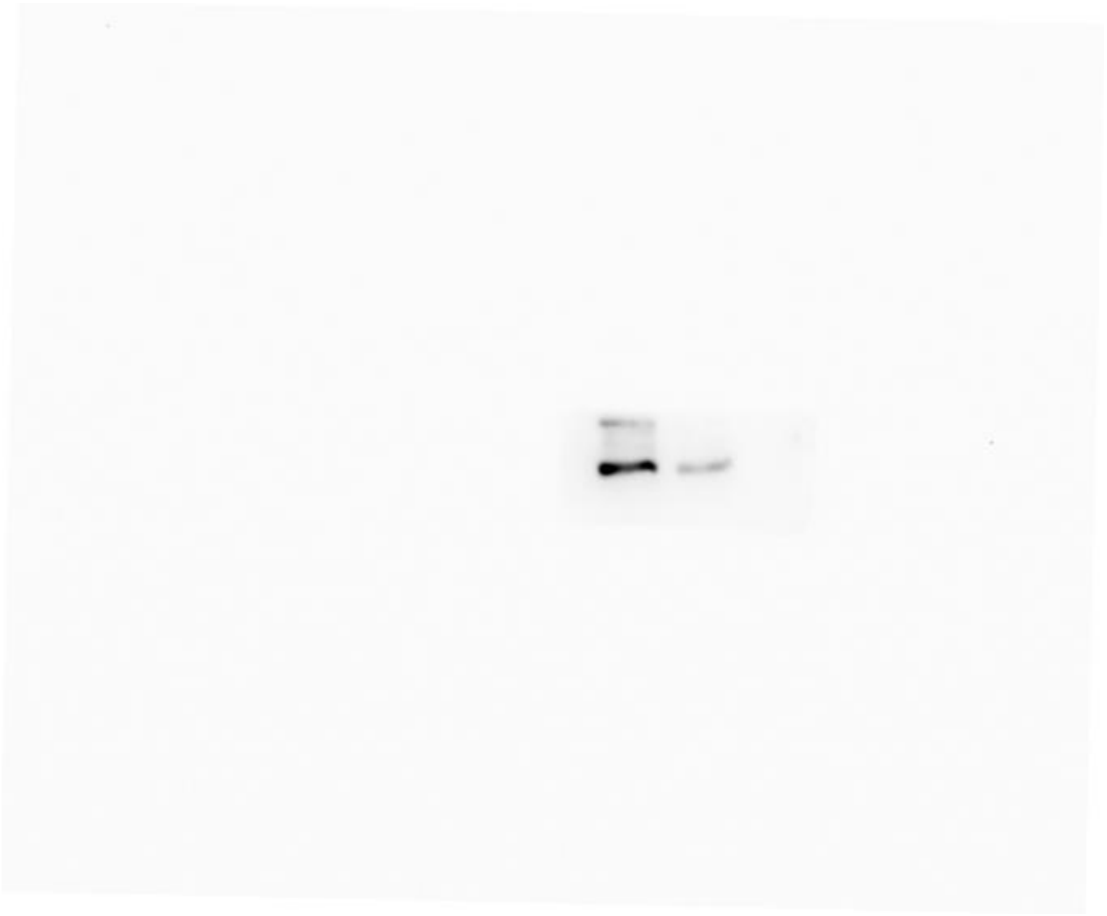

Fig 3E. IB: N-cadherin (SKOV3 cell sample: EV, CRAT)

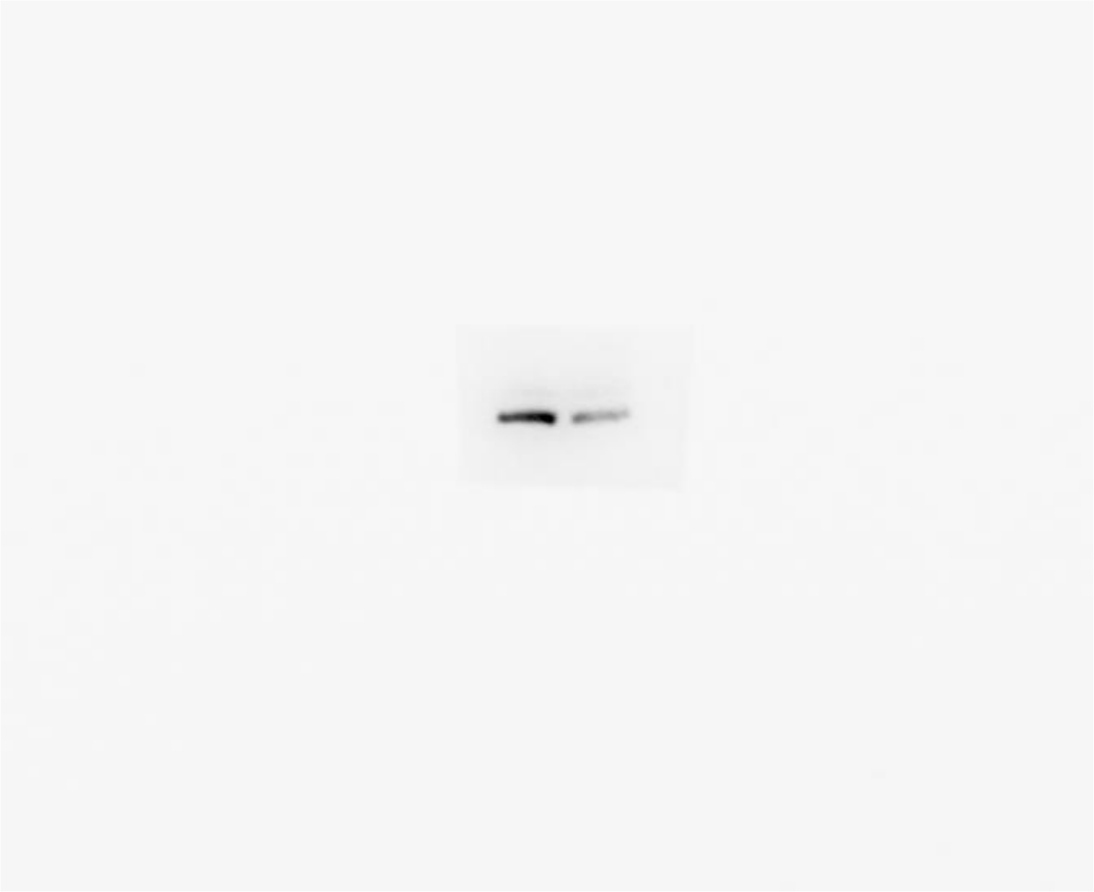

Fig 3E. IB: Vimentin (SKOV3 cell sample: EV, CRAT)

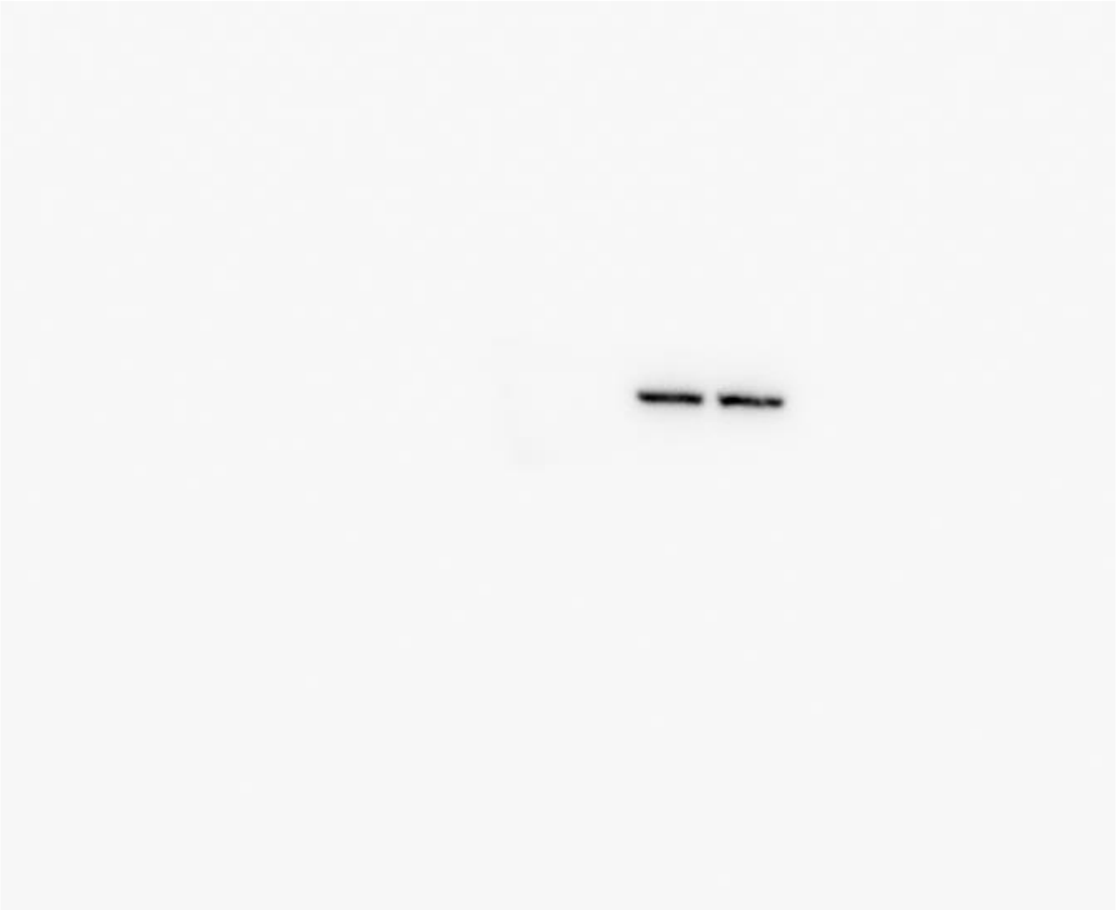

Fig 3E. IB: β-actin (SKOV3 cell sample: EV, CRAT)

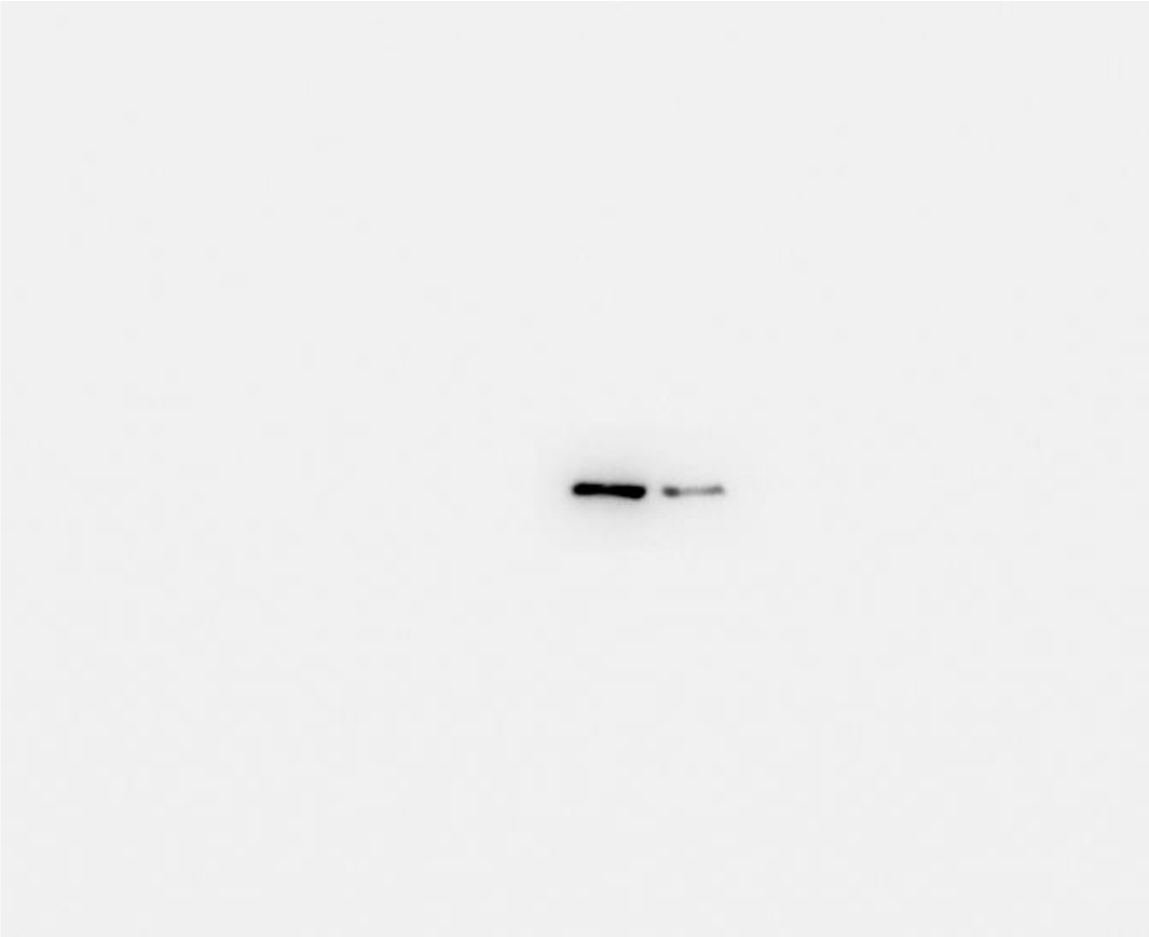

Fig 5B. IB: CRAT (A2780 cell sample: siCtrl, si-CRAT)

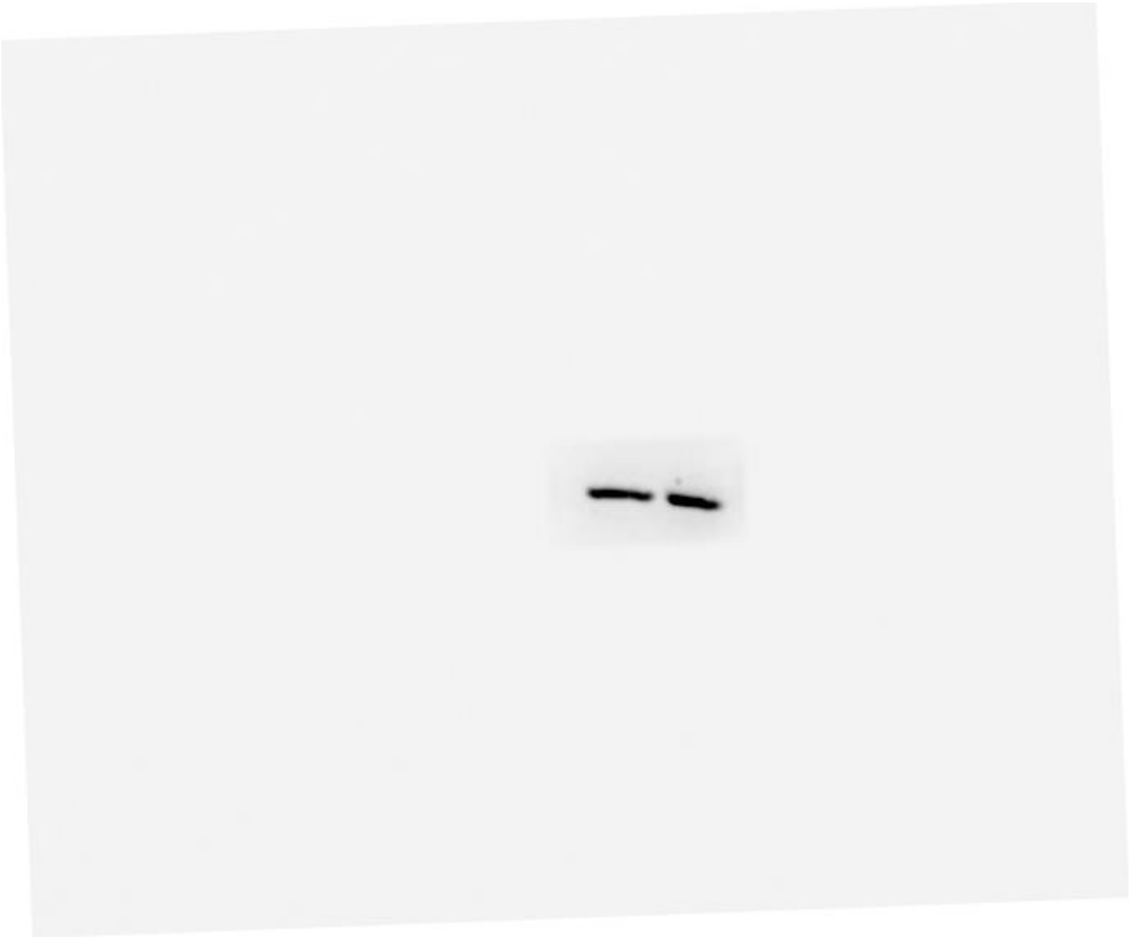

Fig 5B. IB: β-actin (A2780 cell sample: siCtrl, si-CRAT)

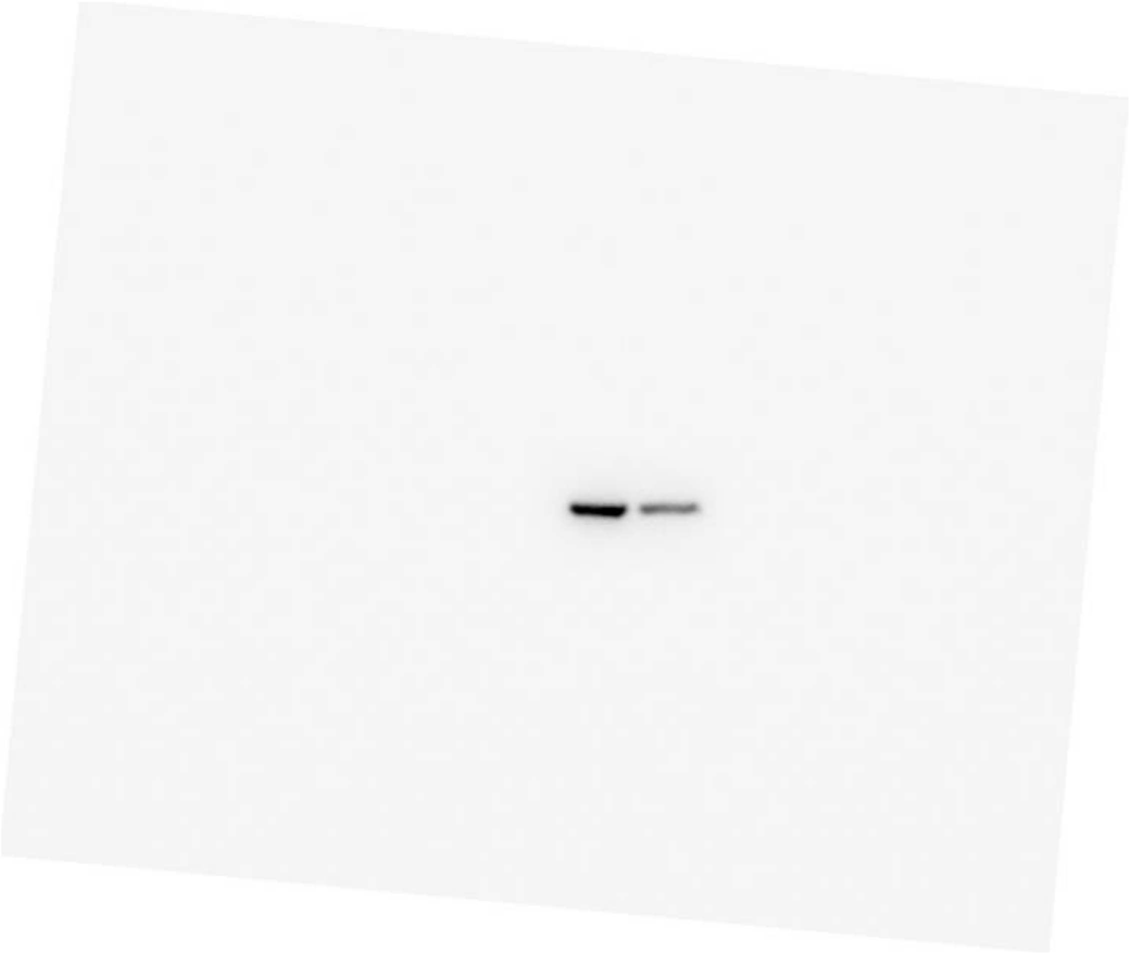

Fig 5B. IB: CRAT (ES2 cell sample: siCtrl, si-CRAT)

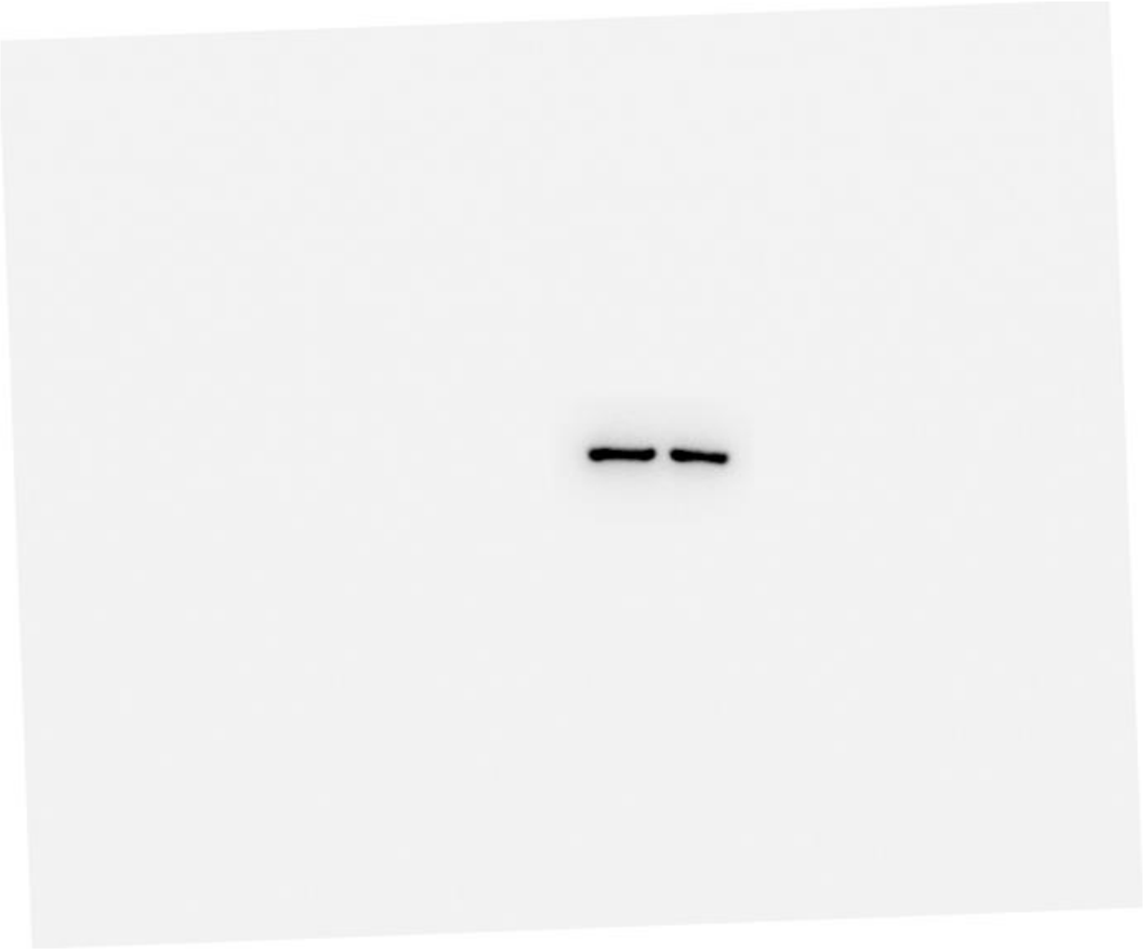

Fig 5B. IB: β-actin (ES2 cell sample: siCtrl, si-CRAT)

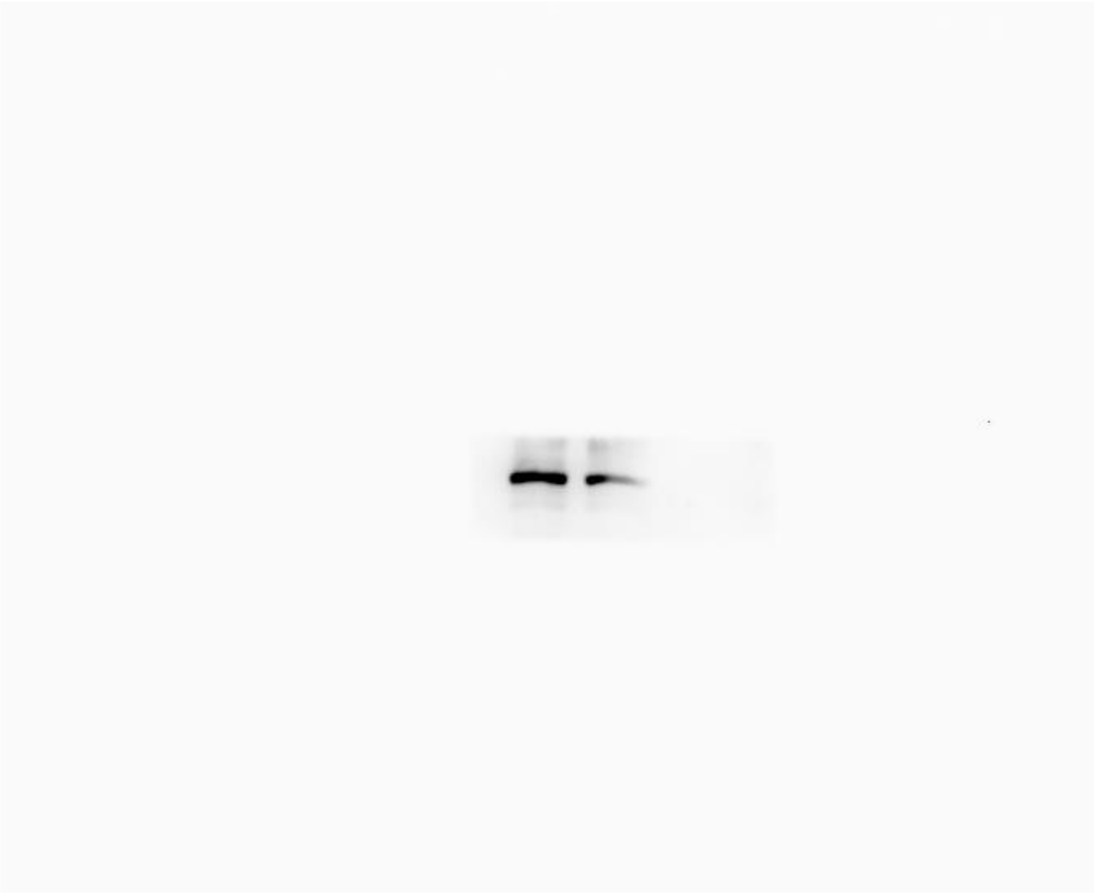

Fig 7C. IB: PGC1-α (HEY cell sample: EV, CRAT)

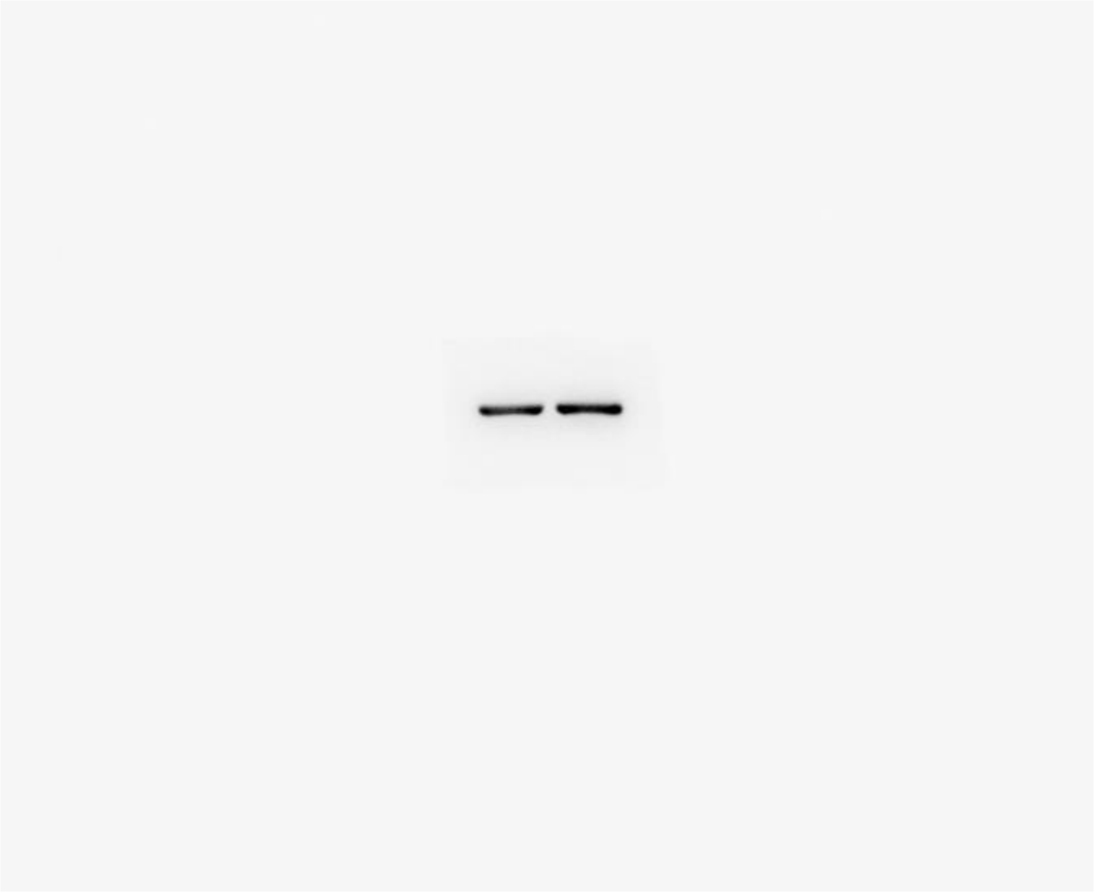

Fig 7C. IB: β-actin (HEY cell sample: EV, CRAT)

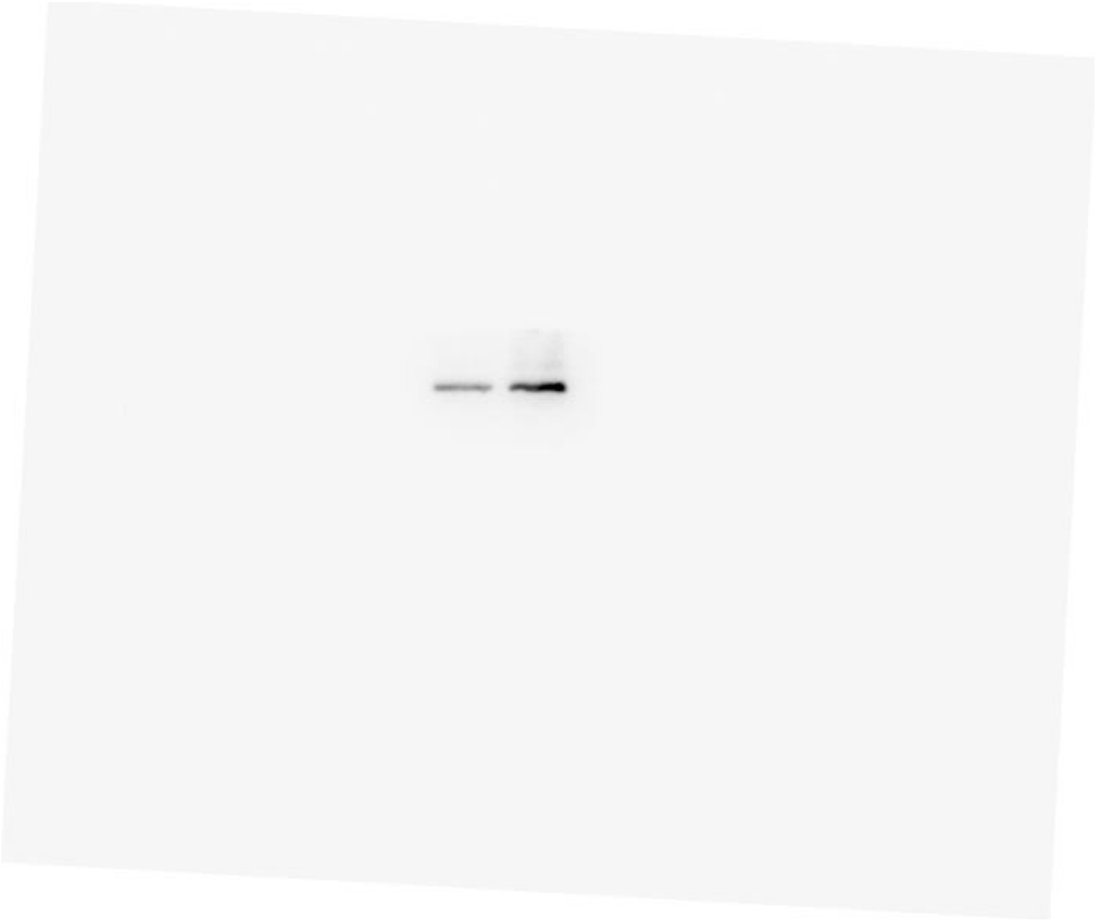

Fig 7C. IB: PGC1-α (A2780 cell sample: shCtrl, shCRAT)

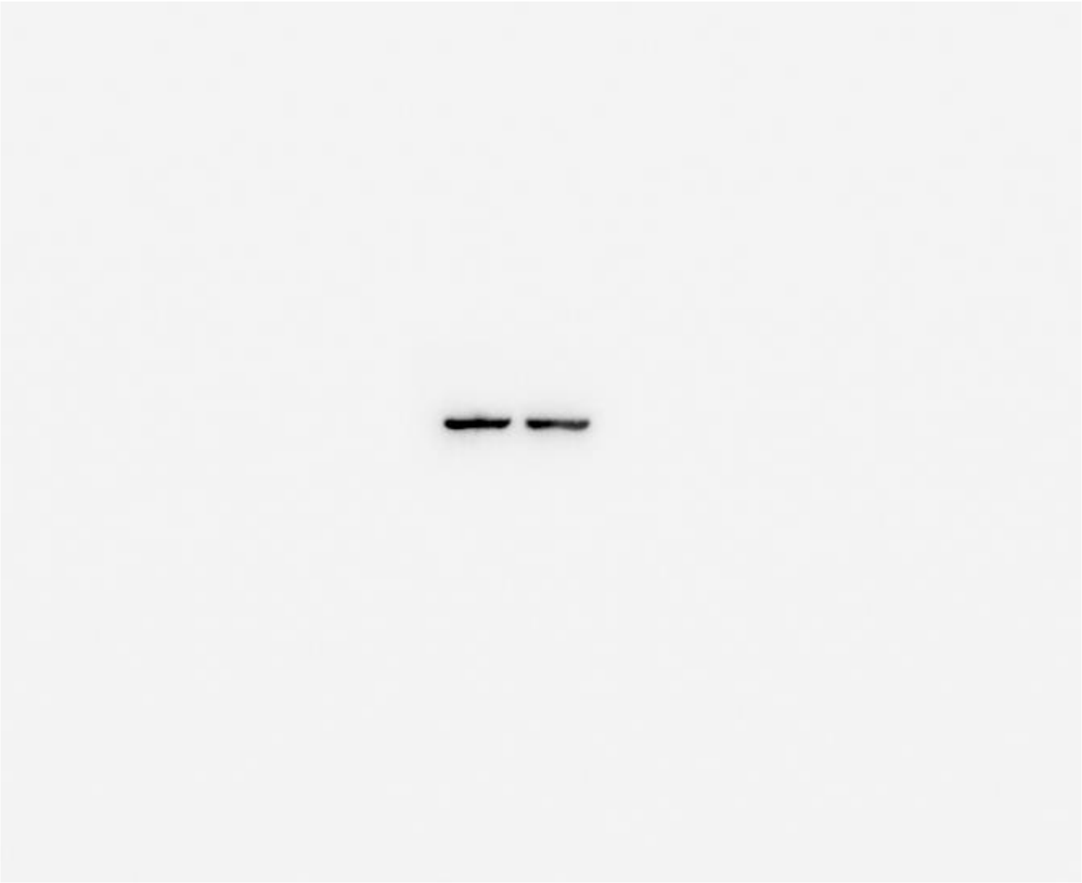

Fig 7C. IB: β-actin (A2780 cell sample: shCtrl, shCRAT)

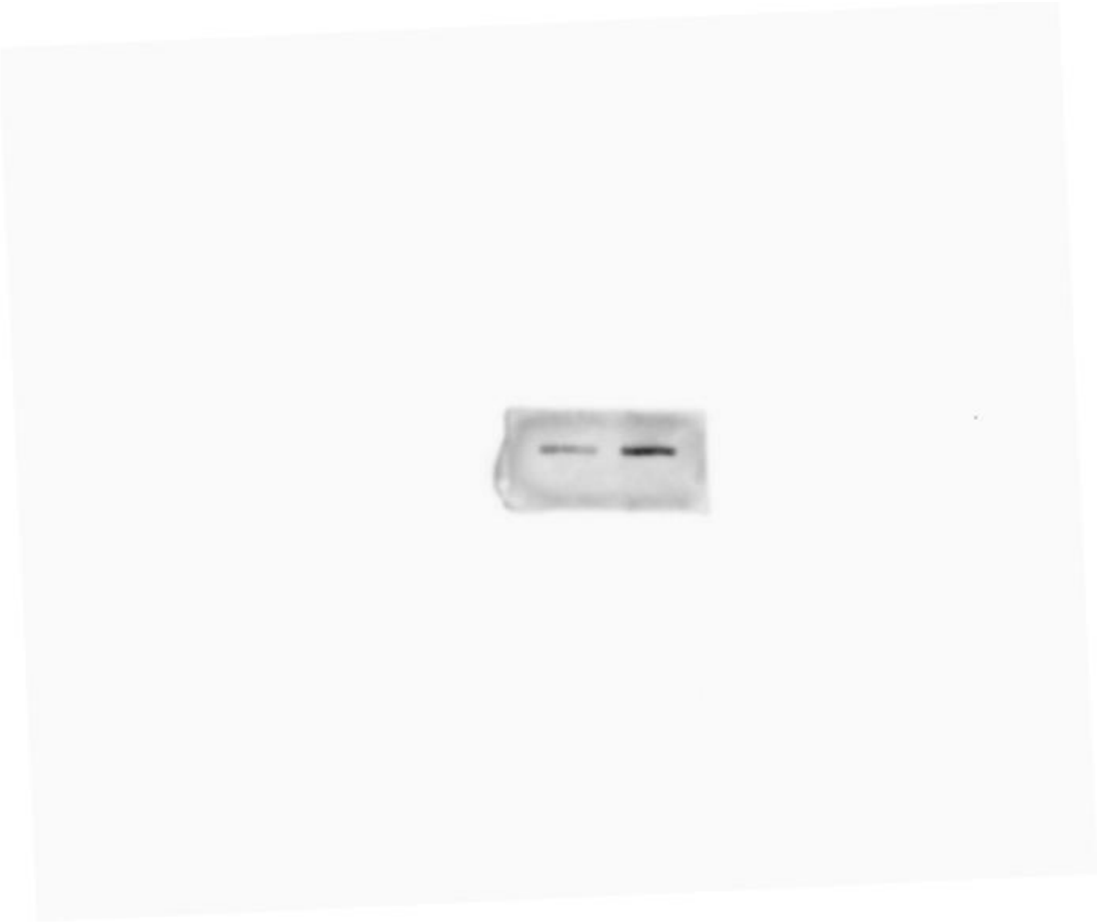

Fig 7E. IB: Ac-K (HEY cell sample: EV, CRAT)

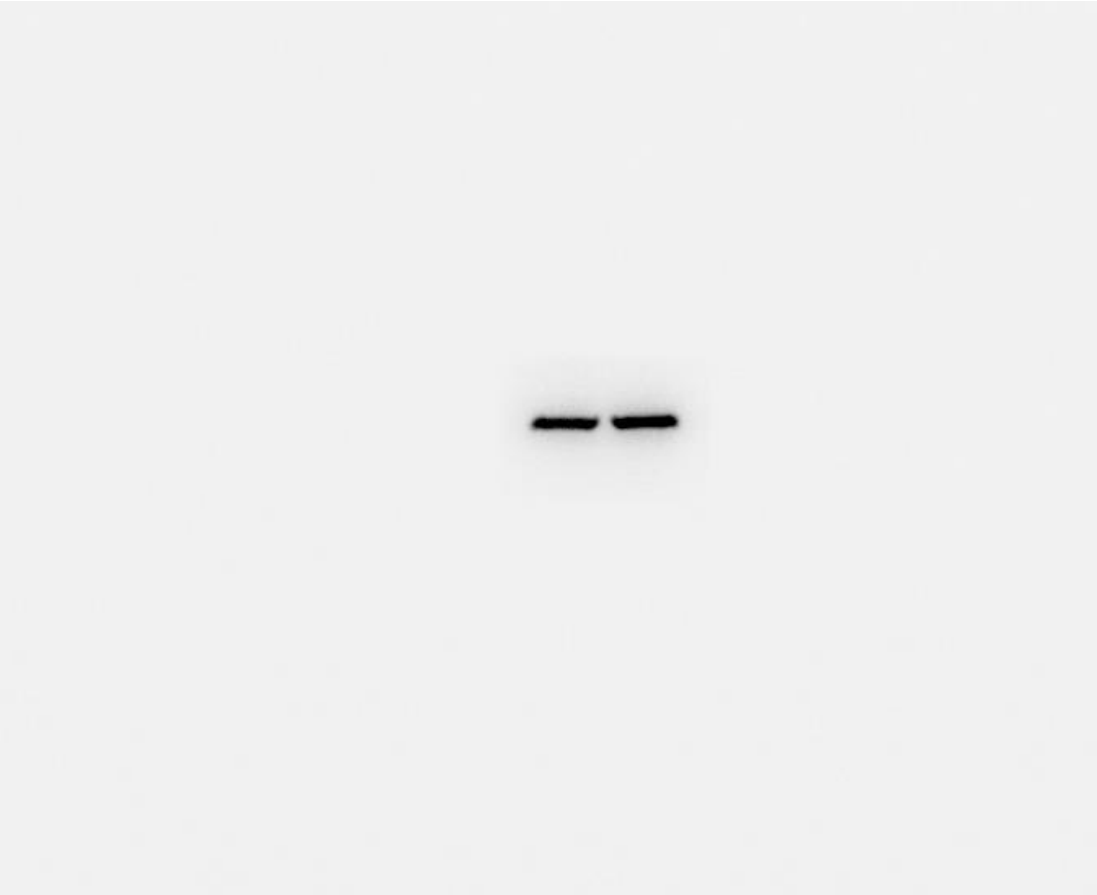

Fig 7E. IB: PGC1-α (HEY cell sample: EV, CRAT)

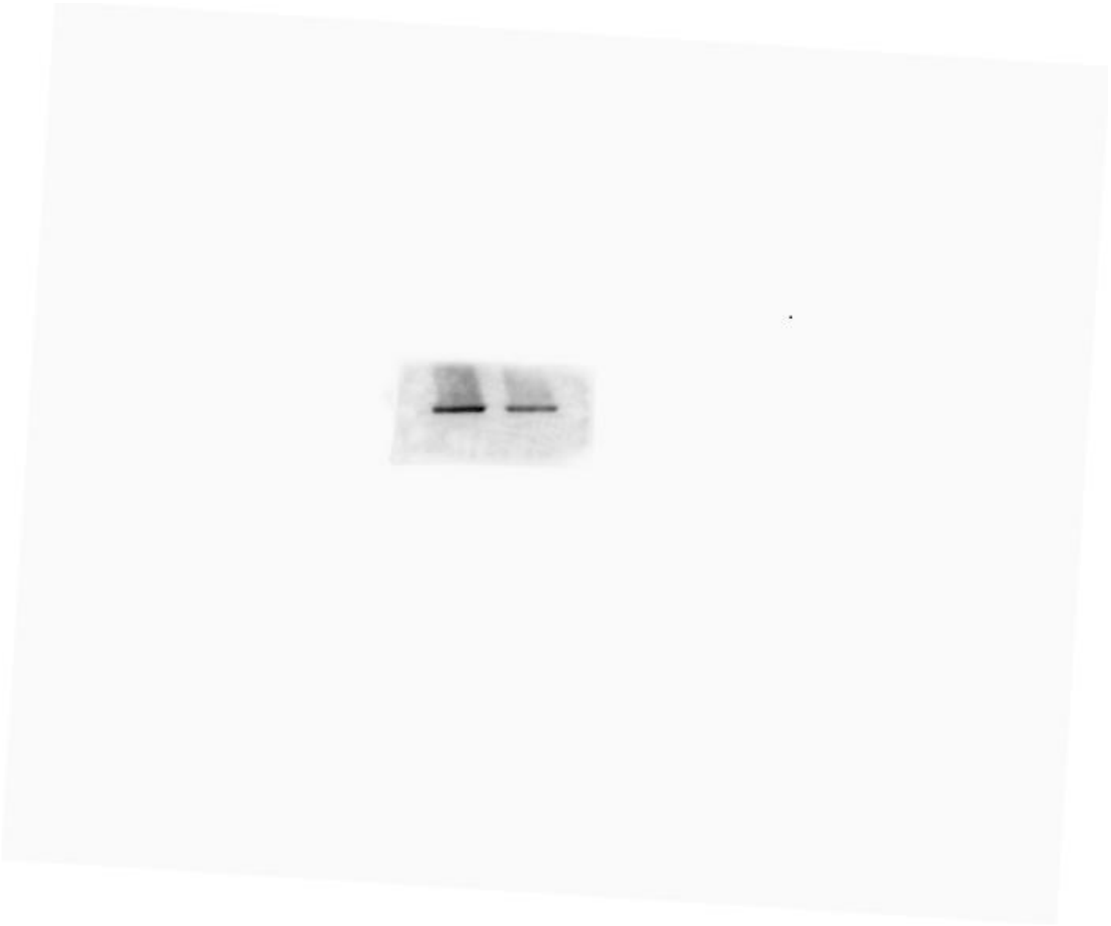

Fig 7E. IB: Ac-K (A2780 cell sample: shCtrl, shCRAT)

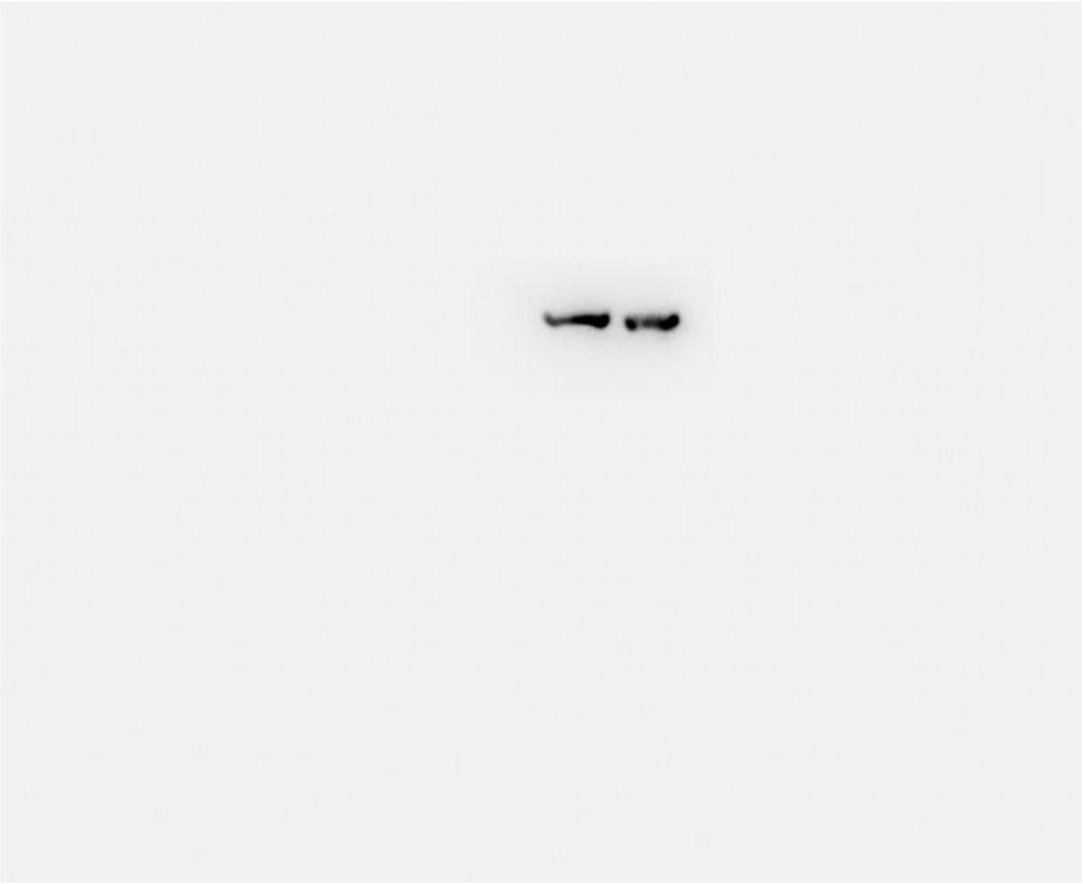

Fig 7E. IB: PGC1-α (A2780 cell sample: shCtrl, shCRAT)

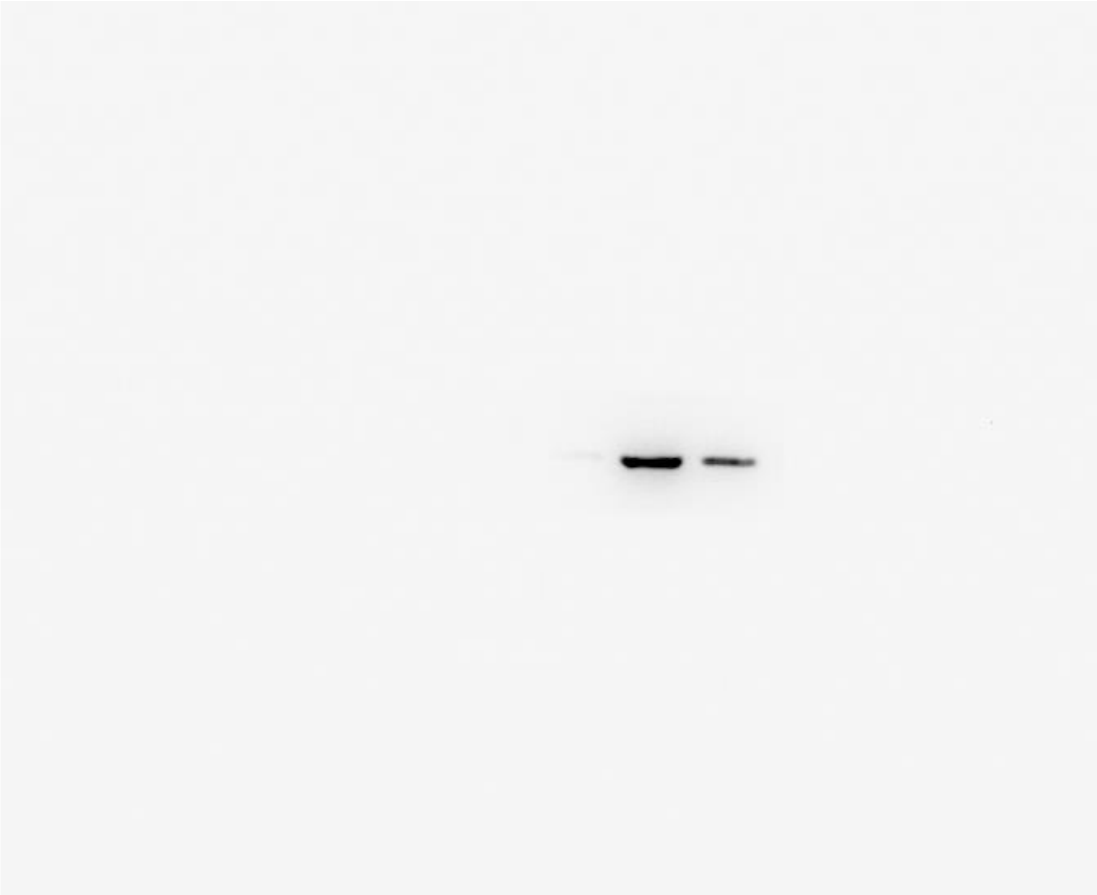

Fig 9B. IB: CRAT (A2780 cell sample: Ctrl, miR-132-5p)

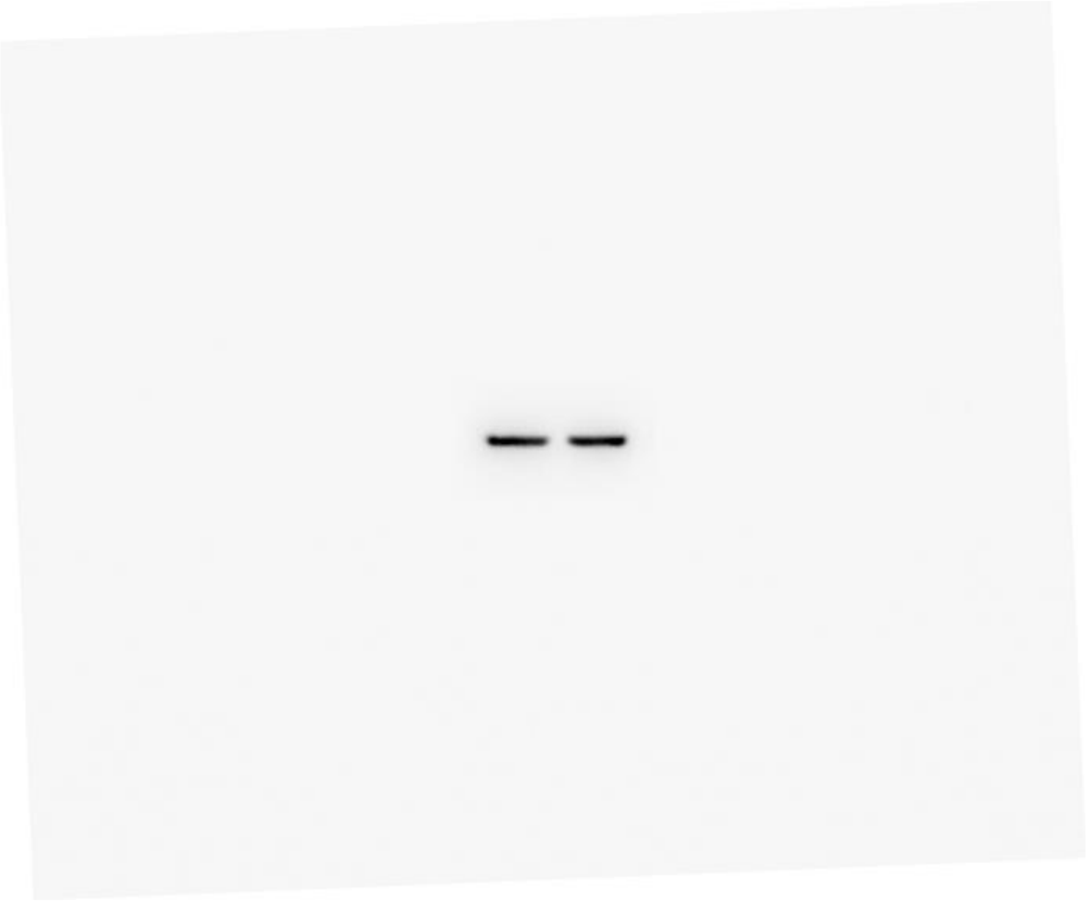

Fig 9B. IB: β-actin (A2780 cell sample: Ctrl, miR-132-5p)

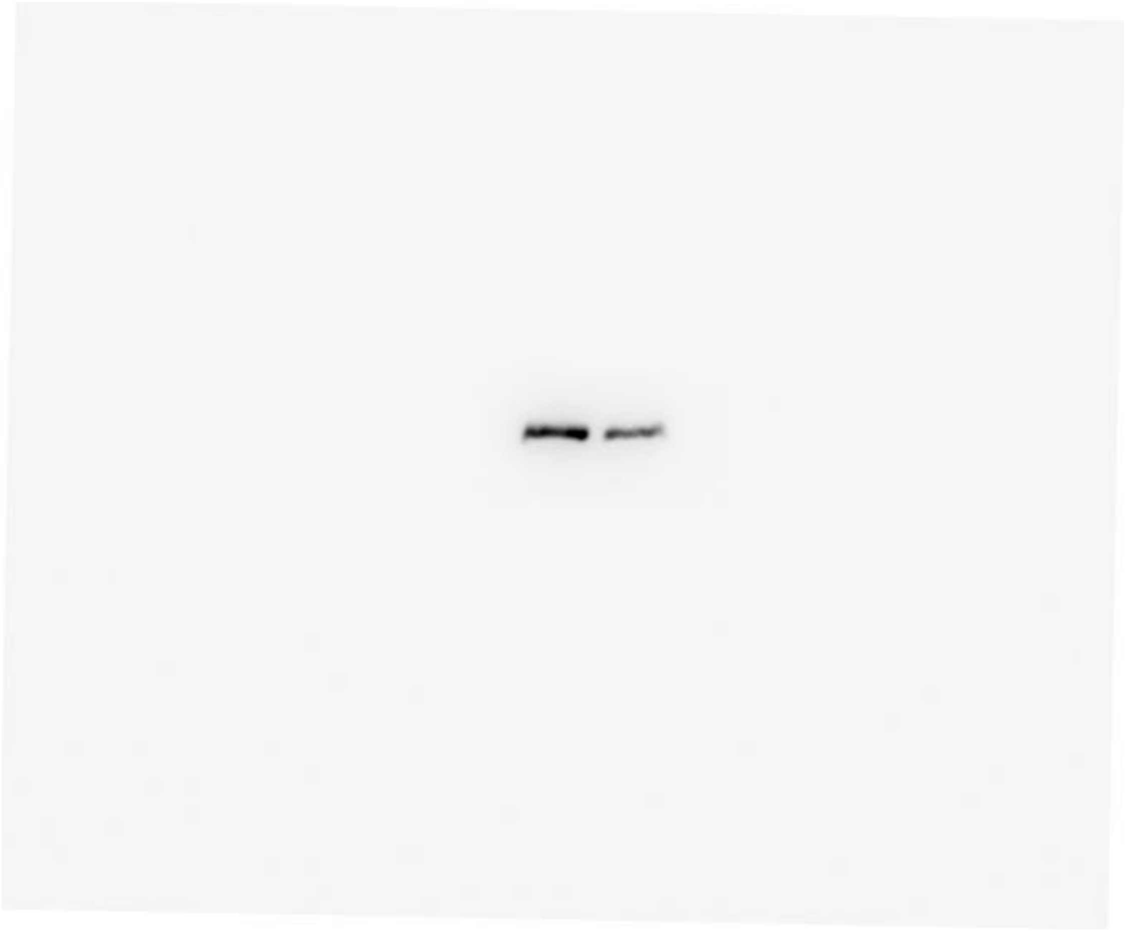

Fig 9B. IB: CRAT (ES2 cell sample: Ctrl, miR-132-5p)

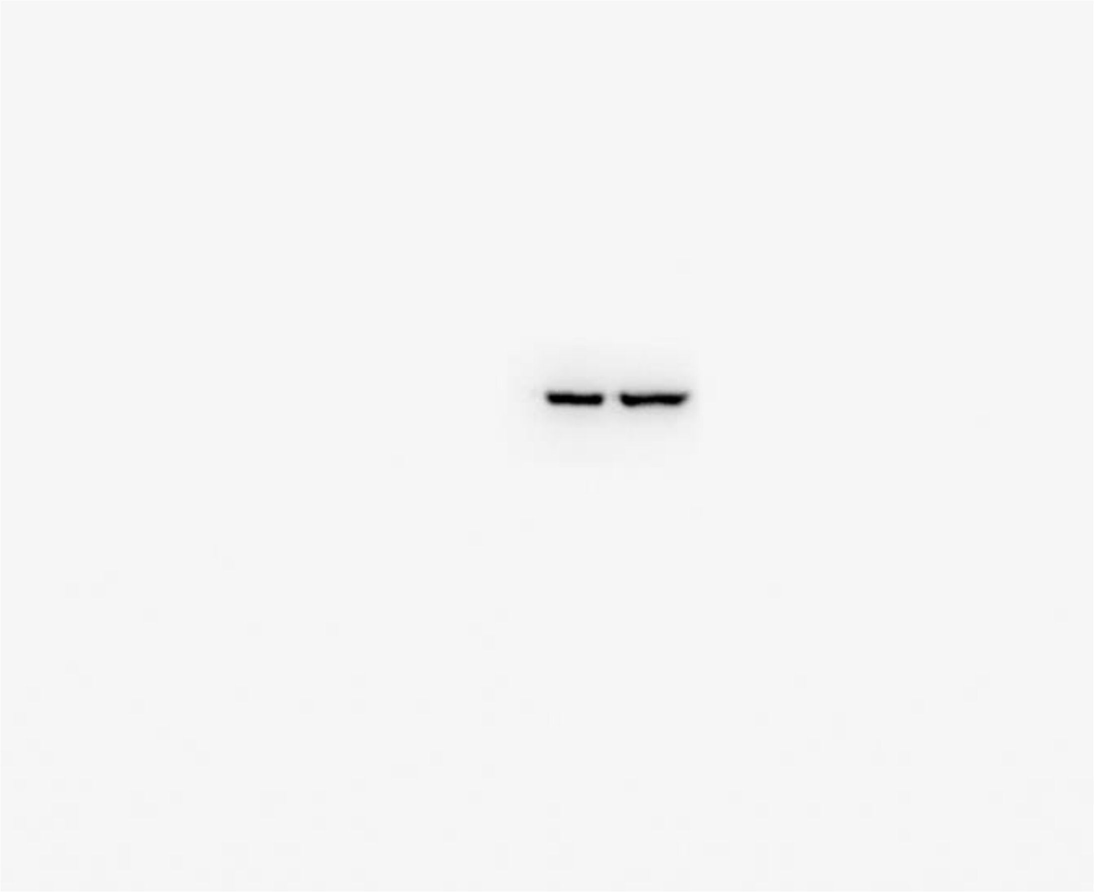

Fig 9B. IB: β-actin (ES2 cell sample: Ctrl, miR-132-5p)

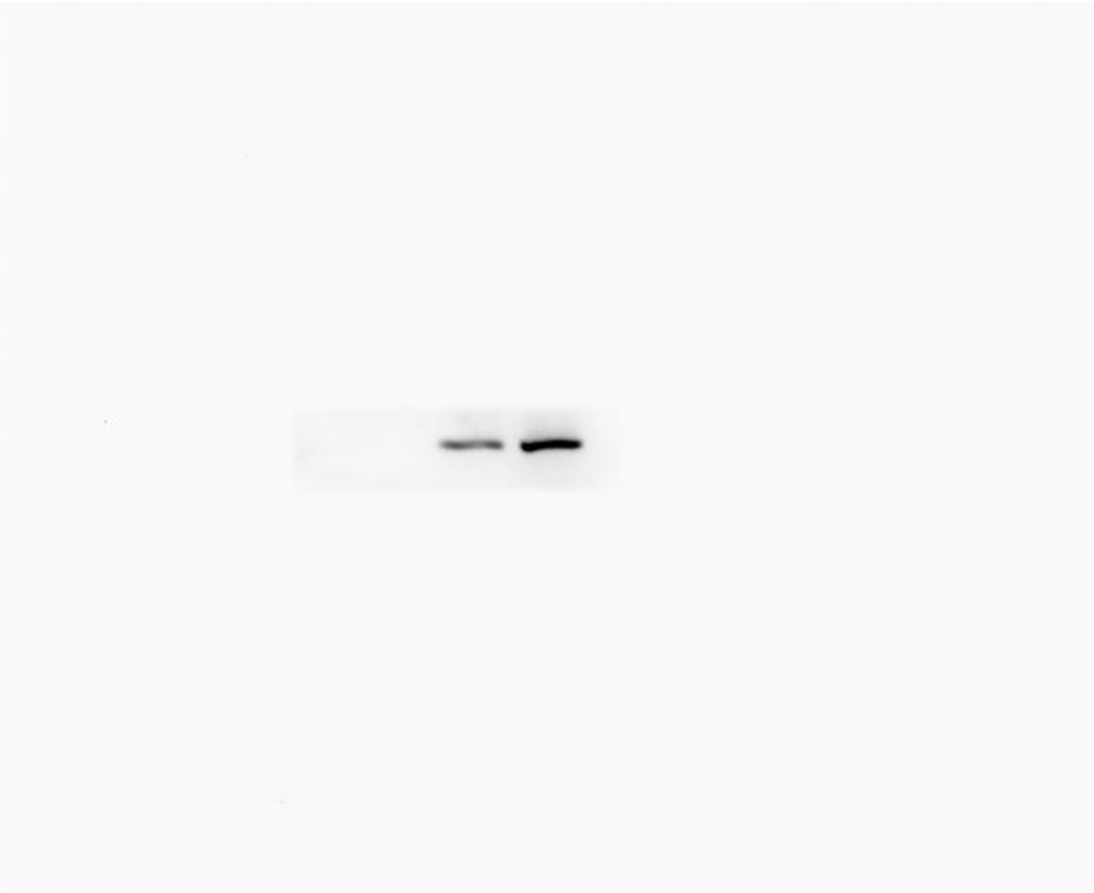

Fig S3B. IB: CRAT (ES2 cell sample: EV, CRAT)

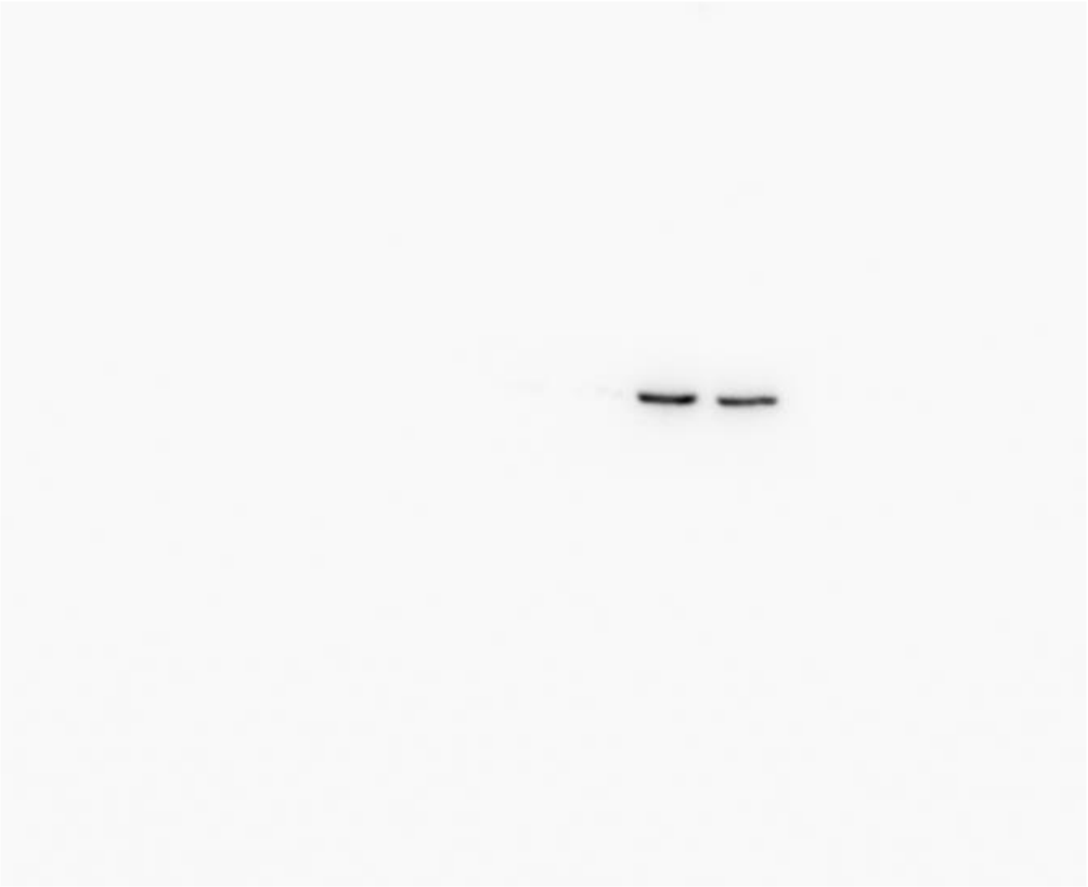

Fig S3B. IB: β-actin (ES2 cell sample: EV, CRAT)
